# Predictive, preventive, and personalized medicine in breast cancer: targeting the PI3K pathway

**DOI:** 10.1186/s12967-023-04841-w

**Published:** 2024-01-03

**Authors:** Muhammad Tufail, Jia-Ju Hu, Jie Liang, Cai-Yun He, Wen-Dong Wan, Yu-Qi Huang, Can-Hua Jiang, Hong Wu, Ning Li

**Affiliations:** 1 Department of Oral and Maxillofacial Surgery, Center of Stomatology, Xiangya Hospital, Central South University, Changsha, China; 2 Institute of Oral Precancerous Lesions, Central South University, Changsha, China; 3 Research Center of Oral and Maxillofacial Tumor, Xiangya Hospital, Central South University, Changsha, China; 4 National Clinical Research Center for Geriatric Disorders, Xiangya Hospital, Central South University, Changsha, China; 5 State Key Laboratory of Powder Metallurgy, Central South University, Changsha, 410083 China

**Keywords:** Breast cancer, PI3K pathway, Predictive medicine, Preventive medicine, Personalized medicine

## Abstract

Breast cancer (BC) is a multifaceted disease characterized by distinct molecular subtypes and varying responses to treatment. In BC, the phosphatidylinositol 3-kinase (PI3K) pathway has emerged as a crucial contributor to the development, advancement, and resistance to treatment. This review article explores the implications of the PI3K pathway in predictive, preventive, and personalized medicine for BC. It emphasizes the identification of predictive biomarkers, such as PIK3CA mutations, and the utility of molecular profiling in guiding treatment decisions. The review also discusses the potential of targeting the PI3K pathway for preventive strategies and the customization of therapy based on tumor stage, molecular subtypes, and genetic alterations. Overcoming resistance to PI3K inhibitors and exploring combination therapies are addressed as important considerations. While this field holds promise in improving patient outcomes, further research and clinical trials are needed to validate these approaches and translate them into clinical practice.

**Graphical Abstract:**

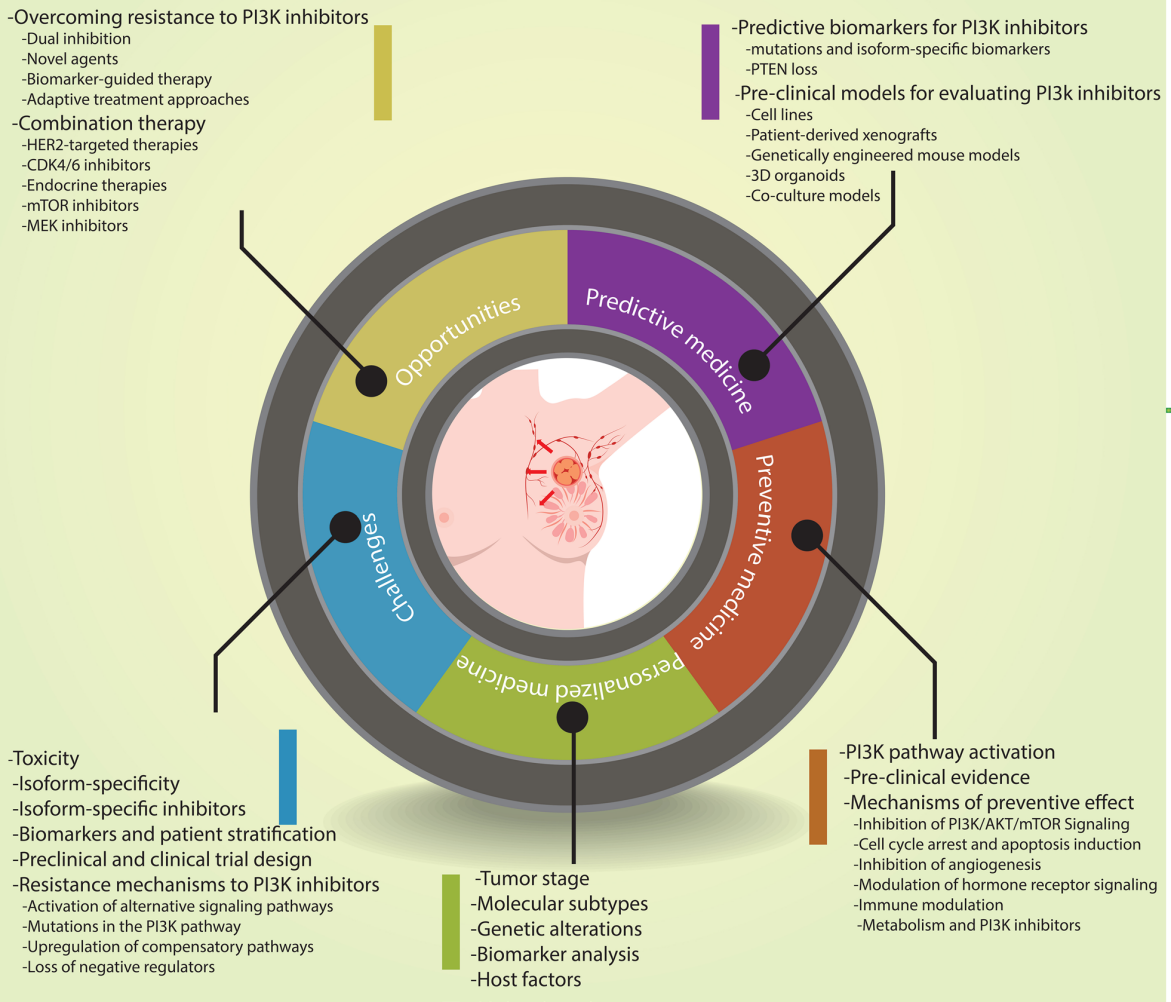

## Introduction

Breast cancer stands as the prevailing form of cancer among women worldwide, exhibiting the highest incidence rate compared to other types of cancer [1, 2]. This complex and diverse disease is characterized by uncontrolled proliferation and growth of abnormal cells in breast tissue. There are diverse subtypes of BC, each with diverse clinical and molecular features and requiring individualized approaches for diagnosis and treatment [3, 4]. Despite advances in oncology, BC remains a global health challenge, requiring further research and innovative therapies [5].

The PI3K pathway has emerged as a key player in BC development [6, 7]. The PI3K pathway governs a wide range of cellular processes through its signaling mechanism, such as cell growth [8, 9], survival [10, 11], metabolism [8, 12] and motility [13]. Dysregulation of the PI3K pathway is linked with several aspects of BC development and progression [14, 15]. Malactivation of this pathway occurs through various mechanisms, including genetic alterations and somatic mutations [16, 17], gene amplification [18], and loss of negative regulators [19]. Such alterations sustain and reinforce the signaling of the PI3K pathway, resulting in amplified cell proliferation, enhanced cell survival, and the emergence of therapy resistance.

The Downstream activation of the PI3K signaling pathway occurs through multiple receptor tyrosine kinases, among which the epidermal growth factor receptor (EGFR) [20], insulin-like growth factor receptor (IGFR) [21], and human epidermal growth factor receptor 2 (HER2) [22] are included. Upon binding with ligands, these receptors initiate the activation of PI3K, triggering the conversion of phosphatidylinositol 4,5-bisphosphate (PIP2) into phosphatidylinositol 3,4,5-trisphosphate (PIP3) [23]. Acting as a secondary messenger, PIP3 recruits proteins harboring pleckstrin homology domains (PH), such as AKT and PDK1, to the plasma membrane [24]. AKT activation promotes cell survival and proliferation through phosphorylation of downstream targets such as mTOR (mammalian target of rapamycin) [11, 25], BAD [26, 27], and FOXO transcription factors [28, 29].

The significance of PI3K signaling in BC extends to crucial implications in diagnosis, prognosis, and treatment. Targeting this pathway shows promise in overcoming resistance and enhancing outcomes. Nevertheless, resistance mechanisms pose limitations to the clinical effectiveness of inhibitors targeting PI3K, AKT, and mTOR. This comprehensive review emphasizes the pivotal role of the PI3K pathway in BC, delving into its dysregulation, impact on disease progression, and development of therapy resistance. It discusses current strategies, challenges in clinical translation, and advancements in prediction, prevention, and personalized medicine. The article emphasizes the significance of tailored therapies and predictive biomarkers to improve BC patients' outcomes and quality of life.

## PI3K isoforms and classes

**Fig. 1:**
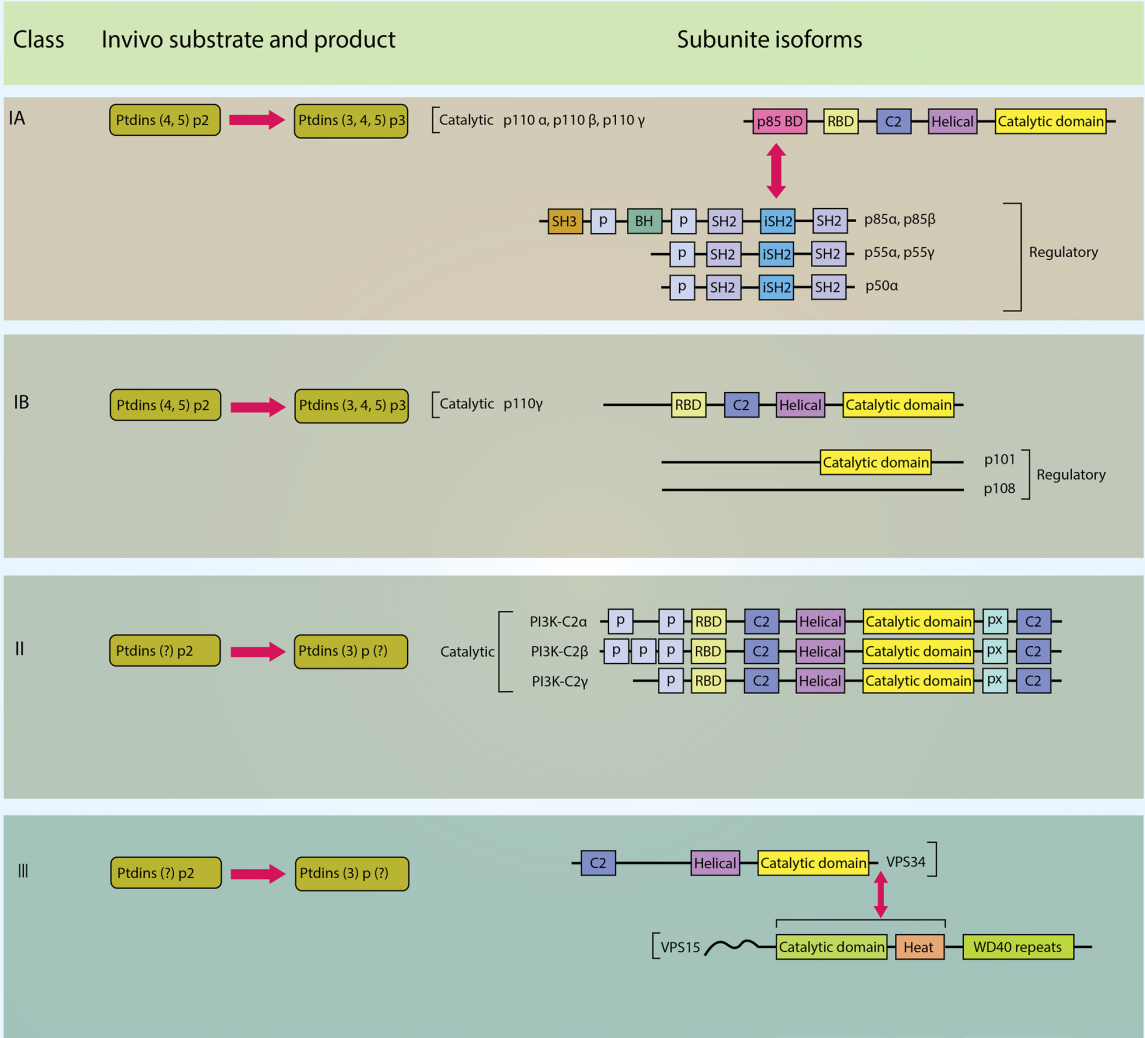
PI3Ks are classified based on their substrate specificities and structures. Class IA and IB PI3Ks phosphorylate PtdIns(4,5)P2, while class III PI3Ks phosphorylate PtdIns. Class II PI3Ks may also preferentially phosphorylate PtdIns. Class IA PI3Ks consist of p110 catalytic subunits and p85 regulatory subunits, with specific domains such as p85-BD, RBD, helical, and catalytic domains. Class IB PI3Ks comprise p110γ catalytic subunits and p101 or p87 regulatory subunits. Class II PI3Ks, including PI3K-C2α, PI3K-C2β, and PI3K-C2γ, have monomeric structures. The only class III PI3K is VPS34, which forms a heterodimer with VPS15 and possesses helical and catalytic domains. Various domains, such as P domains and C2 domains, are also indicated in their structures

In mammals, PI3Ks can be categorized into three classes. Class I PI3K can be further subdivided into subclasses IA and IB based on their regulation. Class IA PI3Ks comprise the catalytic subunits p110 and the regulatory subunit p85. The genes PIK3CA, PIK3CB, and PIK3CD encode the catalytic isoforms p110α, p110β, and p110δ, respectively. These isoforms can be associated with various regulatory isoforms, including p85α, p85β, and p55γ, collectively called p85-type regulatory subunits. The genes PIK3R1, PIK3R2, and PIK3R3 encode these regulatory subunits. On the other hand, class IB PI3Ks interact with the catalytic subunit p110γ, encoded by PIK3CG, and the regulatory isoforms p101 (encoded by PIK3R5) or p87 (also known as p84 or p87PIKAP, encoded by PIK3R6). The expression of p110α and p110β is widespread, while p110δ and p110γ are mainly found in leukocytes (Fig. 1) [30].

## Predictive medicine approach to breast cancer

A predictive medicine approach to BC involves identifying and using biomarkers to predict response to specific treatments. For BC, predictive biomarkers help determine the efficacy of PI3K inhibitors. Understanding the predictive biomarkers of PI3K inhibitors is critical for selecting the most appropriate therapy and improving patient outcomes.

### Predictive biomarkers for PI3K inhibitors in breast cancer

Predictive biomarkers are vital in guiding treatment decisions and predicting response to specific BC treatments [31]. Several biomarkers associated with PI3K inhibitors have been identified that may help predict the efficacy of these inhibitors in breast cancer patients [32, 33]. In particular, the PI3K/AKT/mTOR signaling pathway has been extensively studied due to its dysregulation and availability of targeted inhibitors in BC, particularly PIK3CA mutation and PTEN loss [34, 35].

#### PIK3CA mutations, activation of downstream targets and PI3K isoform-specific biomarkers

The PIK3CA gene, which encodes the p110α catalytic subunit of PI3K [36, 37], is frequently mutated in BC [38, 39]. Studies have shown that patients with PIK3CA mutations tend to have increased activation of PI3K pathway and may have increased sensitivity to PI3K inhibitors [38]. Identifying PIK3CA mutations through testing can assist in determining the potential effectiveness of PI3K inhibitor therapy for patients.

Several biomarkers tested in tumor tissue samples provide additional information on PI3K pathway activation and response to PI3K inhibitors. These include markers such as phosphorylated AKT (pAKT) [40], and phosphorylated S6 (pS6) [41] that indicate activation of the PI3K pathway.

PI3K inhibitors can target different isoforms of the PI3K enzyme [42]. A biomarker that indicates the presence or activation of a particular isoform will help select the most appropriate PI3K inhibitor for a particular patient. For example, PIK3CA mutations are more commonly linked with sensitivity to PI3Kα inhibitors.

Liquid biopsy, which analyzes circulating tumor DNA (ctDNA) or circulating tumor cells (CTCs) in blood [43, 44], provides a non-invasive approach to assess PIK3CA mutations and monitor treatment response [45, 46]. Detection of PIK3CA mutations in ctDNA or CTCs may help select patients for PI3K inhibitor therapy.

Next-generation sequencing, such as genomic profiling, enables comprehensive analysis of the genetic landscape of breast cancer [47]. This approach allows us to identify additional genetic alterations that may affect response to PI3K inhibitors, including alterations in other genes involved in PI3K signaling and genes associated with therapy resistance.

It is important to note that although these biomarkers are associated with response to PI3K inhibitors, they do not conclusively predict individual patient outcome. Further validation with clinical trials and real-world data is needed to optimize patient selection for PI3K inhibitor therapy. Ongoing research aims to identify additional biomarkers and develop more accurate predictive models for personalized BC treatment.

#### PTEN loss

The PTEN gene, short for phosphatase and tensin homolog, is pivotal in suppressing tumors. It aids in regulating the PI3K/AKT pathway, which is crucial for maintaining cellular functionality and preventing abnormal cell proliferation [48]. This pathway is complicated in the control of some cellular processes such as cell growth [49, 50], survival [51] and metabolism [52]. The PTEN gene acts as a negative PI3K/AKT pathway regulator, inhibiting its activation and maintaining cellular homeostasis [53, 54].

Loss of PTEN expression or PTEN gene mutation, another biomarker, is linked with enhanced activation of the PI3K pathway and potential susceptibility to PI3K inhibitors [55]. Moreover, as PIK3CA mutations are more common in ER+ BC [56], hormone receptor status, particularly estrogen receptor (ER) positivity, is associated with the potential efficacy of PI3K inhibitors in this subtype.

Loss of PTEN function due to genetic mutation or epigenetic silencing disrupts the PI3K/AKT pathway, leading to increased activation and tumor formation [57]. The dysregulation of PTEN can result in uncontrolled cell proliferation and survival, key factors in the development and progression of cancer. Specifically, BC patients who have experienced loss of PTEN function tend to exhibit a greater potential for therapeutic benefits from PI3K inhibitors [58]. By specifically targeting the overactive HER PI3K/AKT pathway, these inhibitors could restore the balance of cellular processes and potentially inhibit cancer cell proliferation and metastasis.

#### AKT1

AKT1 (v-AKT Murine Thymoma Viral Oncogene Homolog 1) has emerged as a prospective predictive biomarker in breast cancer, offering valuable insights into disease prognosis and treatment response. Beyond its role as a signaling molecule, AKT1 functions as a genomic marker, facilitating the tailoring of treatment strategies for individual patients [31]. The identification of specific genetic alterations within the AKT1 gene, particularly within the pleckstrin homology domain (PH), has been substantiated in breast cancer cases. These somatic mutations lead to the constitutive activation of AKT1 and disrupt the normal regulatory mechanisms governing its activation [59, 60].

Notably, the prevalence of AKT1 mutations is notably higher in specific breast cancer subtypes, particularly HR+ and HER2− tumors [60]. Serving as a predictive biomarker, AKT1 mutations exert an influence on disease prognosis. Studies indicate that breast cancer patients harboring AKT1 mutations may exhibit distinct clinical features and outcomes. These mutations correlate with specific clinicopathological features such as tumor size, grade, and lymph node metastasis, thereby providing additional information for stratifying patients based on their risk profiles [61, 62].

Moreover, investigations into AKT1 mutations aim to predict responses to targeted therapies within the PI3K/AKT/mTOR signaling pathway, where AKT1 assumes a central role [31, 59]. Understanding AKT1 mutation status enables the identification of patients who may benefit from targeted inhibitors of the PI3K pathway [63, 64]. This personalized treatment approach aligns with the principles of precision medicine, allowing the selection of potentially effective treatments based on specific genetic changes in a patient's tumor.

Clinical studies evaluating the efficacy of AKT inhibitors in breast cancer patients with AKT1 mutations underscore the role of AKT1 as a predictive biomarker. These studies seek to assess the safety and efficacy of targeted therapies that directly inhibit the aberrant signaling caused by AKT1 mutations. If successful, these inhibitors hold promise as a treatment option for certain breast cancer patients with AKT1-mutated tumors.

#### PIK3R1

Phosphoinositide 3-kinase regulatory subunit 1 (PIK3R1) serves as the regulatory subunit of the PI3K, a pivotal player in cell signaling pathways governing growth, survival, and metabolism. PIK3R1 plays a crucial role in modulating the activity of the catalytic subunit of PI3K, thereby exerting influence over downstream signaling events [65].

In the realm of breast cancer, PIK3R1 has emerged as a prospective predictive biomarker, offering valuable insights into disease prognosis and treatment responsiveness. Genetic alterations in PIK3R1, encompassing both somatic mutations and copy number variations, have been discerned in breast cancer. These alterations have the potential to induce dysregulation within the PI3K signaling pathway, contributing to unbridled cellular proliferation and enhanced survival capabilities [65].

Of significance is the association of PIK3R1 mutations with specific breast cancer subtypes, suggesting potential implications for disease stratification [66]. As a predictive biomarker, PIK3R1 mutations have been linked to diverse clinical outcomes in breast cancer patients [65]. Investigations have delved into the correlation between PIK3R1 status and various clinicopathological features, including tumor grade, hormone receptor status, and HER2 amplification.

An understanding of the specific molecular characteristics of tumors, particularly the presence of PIK3R1 mutations, holds promise for refining prognostic assessments and tailoring treatment strategies. Furthermore, PIK3R1 status assumes importance in predicting responses to targeted therapy [67]. The PI3K signaling pathway, in which PIK3R1 is a critical component, stands out as a crucial therapeutic target in breast cancer [68]. Clinical trials involving PI3K inhibitors have been conducted, and the presence of PIK3R1 mutations may exert an influence on treatment responsiveness.

Identification of breast cancer patients with PIK3R1 mutations presents an opportunity to select individuals poised to derive greater benefit from PI3K pathway inhibitors, aligning with the principles of precision medicine. The study of PIK3R1 as a predictive biomarker extends beyond its individual role. Through a comprehensive analysis encompassing multiple genomic changes within the PI3K pathway, including not only PIK3R1 mutations but also mutations in PIK3CA and PTEN, a more nuanced understanding of the molecular landscape of breast cancer emerges. This integrated approach enhances the ability to predict patient outcomes and make well-informed therapeutic decisions.

#### INPP4B

Inositol polyphosphate 4-phosphatase type II (INPP4B) is a phosphoinositide phosphatase that plays a pivotal role in the regulation of intracellular signaling pathways, particularly the phosphoinositide 3-kinase (PI3K) pathway. Functionally, INPP4B acts as a tumor suppressor by exerting negative regulation on PI3K signaling, a signaling cascade crucial for controlling cell proliferation, survival, and metabolism. In breast cancer, INPP4B has emerged as a potential predictive biomarker, offering valuable insights into disease prognosis and treatment response [69, 70].

Genetic alterations in INPP4B, including mutations and changes in expression levels, have been identified in breast cancer. Particularly noteworthy is the common occurrence of the loss of INPP4B expression in breast tumors, resulting in heightened activity of the PI3K signaling pathway. This dysregulation contributes significantly to the uncontrolled cellular proliferation and survival characteristic of cancer [69].

As a predictive biomarker, the status of INPP4B is associated with clinical outcomes in breast cancer patients. Rigorous studies have explored the correlation between INPP4B expression levels and various clinicopathological features, such as tumor grade, hormone receptor status, and HER2 amplification. The loss of INPP4B expression has been consistently linked to a more aggressive tumor phenotype and poor prognosis, underscoring its potential as a robust prognostic indicator [71].

Moreover, the predictive utility of INPP4B extends to its role in determining the response to specific therapeutic interventions. Preclinical investigations have suggested that breast cancer cells exhibiting reduced INPP4B expression may display heightened sensitivity to selective inhibitors of the PI3K pathway, presenting a promising avenue for targeted therapeutic strategies [72].

The comprehensive research on INPP4B as a predictive biomarker also encompasses understanding its role within the broader molecular landscape of breast cancer [71]. Integrative analyses, including the evaluation of INPP4B alongside other genetic alterations within the PI3K pathway, are poised to contribute to a more nuanced and holistic understanding of the disease's molecular underpinnings. This integrated approach holds significant potential for refining prognostic assessments and tailoring treatment strategies in breast cancer patients.

### Pre-clinical models for evaluating PI3k inhibitors in breast cancer

Pre-clinical models play a crucial role in evaluating the efficacy of PI3K inhibitors in BC before advancing to clinical trials. Here are some commonly used pre-clinical models for studying PI3K inhibitors in BC.

#### Cell lines

**Fig. 2:**
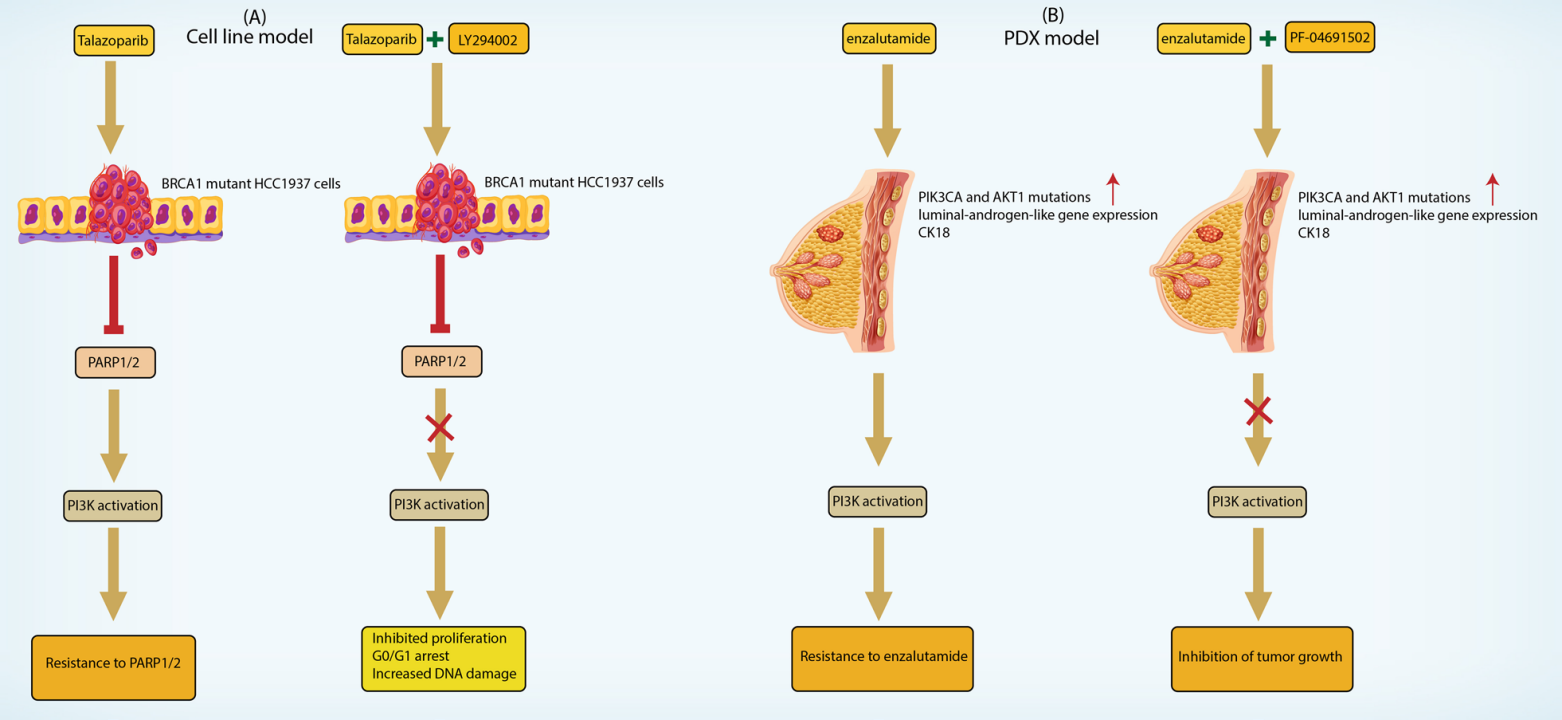
**A** Combined inhibition of Poly (ADP-ribose) polymerase (PARP) by Talazoparib (TAL) and phosphatidylinositol 3-kinase (PI3K) by LY294002 synergistically inhibited the proliferation of BRCA1 mutant HCC1937 triple-negative breast cancer (TNBC) cells. This figure illustrates the enhanced anti-proliferative effects of the combination treatment compared to each drug alone. The combined inhibition induced multiple cellular responses, including apoptosis, G0/G1 arrest, oxidative stress, and increased DNA damage. The synergistic effect suggests that the dual targeting strategy holds promise as a therapeutic approach for TNBC with BRCA1 mutations. **B** LAR (Luminal Androgen Receptor) subtype patient-derived xenografts (PDXs) of triple-negative breast cancer (TNBC) exhibited a significant enrichment of PIK3CA and AKT1 mutations, along with higher levels of luminal-androgen-like gene expression and increased activation of the PI3K pathway proteins, when compared to other TNBC subtypes. Immunohistochemistry analysis revealed strong expression of luminal cytokeratin CK18 and androgen receptor (AR) in three LAR PDX models. In vivo experiments using these PDX models, which harbored genomic alterations of PIK3CA and AKT1 and were resistant to the AR antagonist enzalutamide, demonstrated that mTOR and PI3K inhibitors displayed notable antitumor activity

Cell lines have emerged as essential preclinical models for evaluating the efficacy of PI3K inhibitors in BC research (Fig. 2A) [73, 74]. These models have several advantages (Table 1), including ease of establishment, controlled culture conditions, and high-throughput screening capabilities. The researchers used cell lines to gain valuable insight into the molecular mechanisms of changes in PI3K signaling that commonly occur in BC.

**Table 1:** Advantages and disadvantages of using cell lines as preclinical models

Advantages of cell lines as pre-clinical models	Disadvantages of cell lines as pre-clinical models
Ease of Establishment: Cell lines simplify preclinical model establishment, enabling swift initiation of experiments	Lack of In Vivo Tumor Microenvironment: Cell lines lack the complexity of the in vivo tumor microenvironment, limiting their ability to fully replicate conditions found in patient tumors
Controlled Culture Conditions: Cell lines offer controlled conditions for PI3K inhibitor studies, ensuring consistency and reproducibility	Absence of Interaction with the Immune System: Cell lines lack interaction with the immune system, limiting insights into immune-related responses to PI3K inhibitors in cancer biology
High-Throughput Screening: Cell lines enhance high-throughput screening, allowing rapid testing of various conditions and compounds	Three-Dimensional Structural Complexity: Cell lines lack full three-dimensional structural complexity seen in patient tumors, potentially causing differences in drug response and resistance
Insight into Molecular Mechanisms: Cell lines provide valuable insights into molecular mechanisms of PI3K signaling changes in breast cancer, enhancing understanding of the disease	Translation to Clinical Trials: Cell lines offer valuable preclinical data, but validation in more clinically relevant models like patient-derived xenografts or transgenic mouse models is essential before advancing to clinical trials
Manipulation of Gene Expression: Cell lines permit gene expression manipulation through techniques like RNA interference (RNAi) and CRISPR-Cas9, facilitating targeted studies of genes and signaling pathways	Representativeness: Cell lines may not fully represent the heterogeneity of patient tumors, raising concerns about their representativeness. Molecular and phenotypic characteristics may not fully mirror the diversity seen in breast cancer patients
Molecular Characterization: Thorough molecular characterization of cell lines, including PIK3CA gene mutation identification, ensures accurate representation of breast cancer's genetic landscape	
Study of Drug Combinations: Cell lines are valuable for studying PI3K inhibitor combinations, offering insights into synergies and optimizing therapeutic approaches	

The establishment and characterization of BC cell lines is a critical step in preclinical research [75]. Methods to establish these cell lines from patient tumor samples or cell line repositories have been refined over the years [76, 77]. In addition, extensive molecular characterization, including identification of alterations in the PI3K signaling pathway, such as mutations in the PIK3CA gene [78, 79], ensures that the cell lines used in our experiments accurately represent the genetic landscape of BC.

Cell line studies can gain insight into the mechanism of response to PI3K inhibitors [80]. Researchers can manipulate gene expression in these models using techniques such as RNA interference (RNAi) [81], and CRISPR-Cas9 [82, 83], enabling targeted studies of the role of specific genes and signaling pathways. Conducting these studies will enhance our understanding of the molecular mechanisms underlying drug response and resistance, ultimately facilitating the advancement of novel therapeutic approaches and driving progress in the field of medicine.

Cell lines also serve as valuable tools for studying combinations of PI3K inhibitors and other agents [84, 85]. Evaluating drug combinations, such as chemotherapeutic agents and targeted therapies, allows researchers to identify potential synergies and optimize therapeutic approaches. These studies provide important insights into potential combination strategies to improve the efficacy of PI3K inhibitors in BC treatment.

Despite their advantages, it is important to recognize the limitations of cell line models (Table 1). They lack the in vivo tumor microenvironment, interaction with the immune system, and three-dimensional structural complexity observed in patient tumors. Therefore, it is important to evaluate the results of cell line studies using more clinically relevant models. Patient-derived xenografts or transgenic mouse models prior to being placed in clinical trials.

#### Patient-derived xenografts (PDX)

Patient-derived xenografts (PDX) have proven to be a valuable preclinical model for evaluating the efficacy of PI3K inhibitors in BC research (Fig. 2B) [86]. In these models, the patient's own BC tissue is directly transplanted into immunodeficient mice, preserving the heterogeneity and complexity of the original tumor microenvironment.

PDX models provide a more clinically relevant representation of human breast tumors compared to traditional cell line models [87]. PDX models have several advantages (Table 2), such as using the patient's own tissue ensures that the molecular and genetic features of the tumor are preserved, including the presence of alterations in the PI3K signaling pathway that are common in BC patients. This will allow researchers to assess the effects of PI3K inhibitors on tumors that closely resemble the patient's original tumors, increasing the translatability of the results. One of the key advantages of PDX models is their ability to recapitulate the tumor microenvironment [88]. Breast tumors reside in a complex ecosystem composed of stromal cells [89], immune cells [90], and blood vessels [91]. The PDX model retains these elements, allowing the study of tumor-stromal interactions and the effects of PI3K inhibitors on the tumor microenvironment. Assessing the effectiveness of PI3K inhibitors holds significant relevance, given the crucial involvement of the PI3K signaling pathway in mediating communication between tumor cells and their microenvironment. This understanding helps shed light on the interplay between these factors and enables informed evaluation of PI3K inhibitors' efficacy.

**Table 2:** Advantages and disadvantages of using patient-derived xenografts as preclinical models

Advantages of patient-derived xenografts as pre-clinical models	Disadvantages of patient-derived xenografts as pre-clinical models
Preservation of Tumor Heterogeneity: PDX models preserve tumor heterogeneity by directly transplanting the patient's breast cancer tissue into immunodeficient mice, maintaining the complexity of the original tumor microenvironment	Reliance on Immunodeficient Mice: PDX models depend on immunodeficient mice, restricting the study of interactions with the immune system and potentially impacting the assessment of immune-related responses to PI3K inhibitors
Clinical Relevance: PDX models provide a clinically relevant representation of human breast tumors, preserving molecular and genetic features, including PI3K signaling pathway alterations, more effectively than traditional cell line models	Time-Consuming Establishment: Establishing and disseminating PDX models is time-consuming, impacting the speed of preclinical studies and potentially causing delays in obtaining results
Translatability of Results: Using the patient's own tissue enhances result translatability, enabling researchers to assess the effects of PI3K inhibitors on tumors closely resembling the patient's original tumors	Variability in Transplant Success Rates: Transplant success rates for PDX models vary with tumor type and individual patient characteristics, introducing variability in the effectiveness of model establishment
Recapitulation of Tumor Microenvironment: PDX models recapitulate the tumor microenvironment's complexity, allowing the study of tumor-stromal interactions and the effects of PI3K inhibitors on the microenvironment, including stromal cells, immune cells, and blood vessels	
Insights into Tumor Response and Resistance: Longitudinal studies with PDX models provide insights into tumor response and resistance to PI3K inhibitors, aiding in identifying potential predictive biomarkers for personalized therapeutic strategies	

The PDX model also provides a platform to study the mechanisms of tumor response and resistance to PI3K inhibitors [92, 93]. Longitudinal studies can be performed by treating PDX mice with PI3K inhibitor and monitoring tumor growth and molecular changes over time. This enables the discovery of potential predictive biomarkers for response and resistance, which can inform patient stratification and create personalized therapeutic strategies.

It is important to recognize the limitations of PDX models (Table 2), such as the reliance on immunodeficient mice and the time required to establish and disseminate these models. Furthermore, transplant success rates vary with tumor type and individual patient characteristics. Nevertheless, the PDX model remains a valuable tool for the preclinical evaluation of PI3K inhibitors in BC and provides a more clinically relevant predictive model system.

#### Genetically engineered mouse models (GEMMs)

**Fig. 3:**
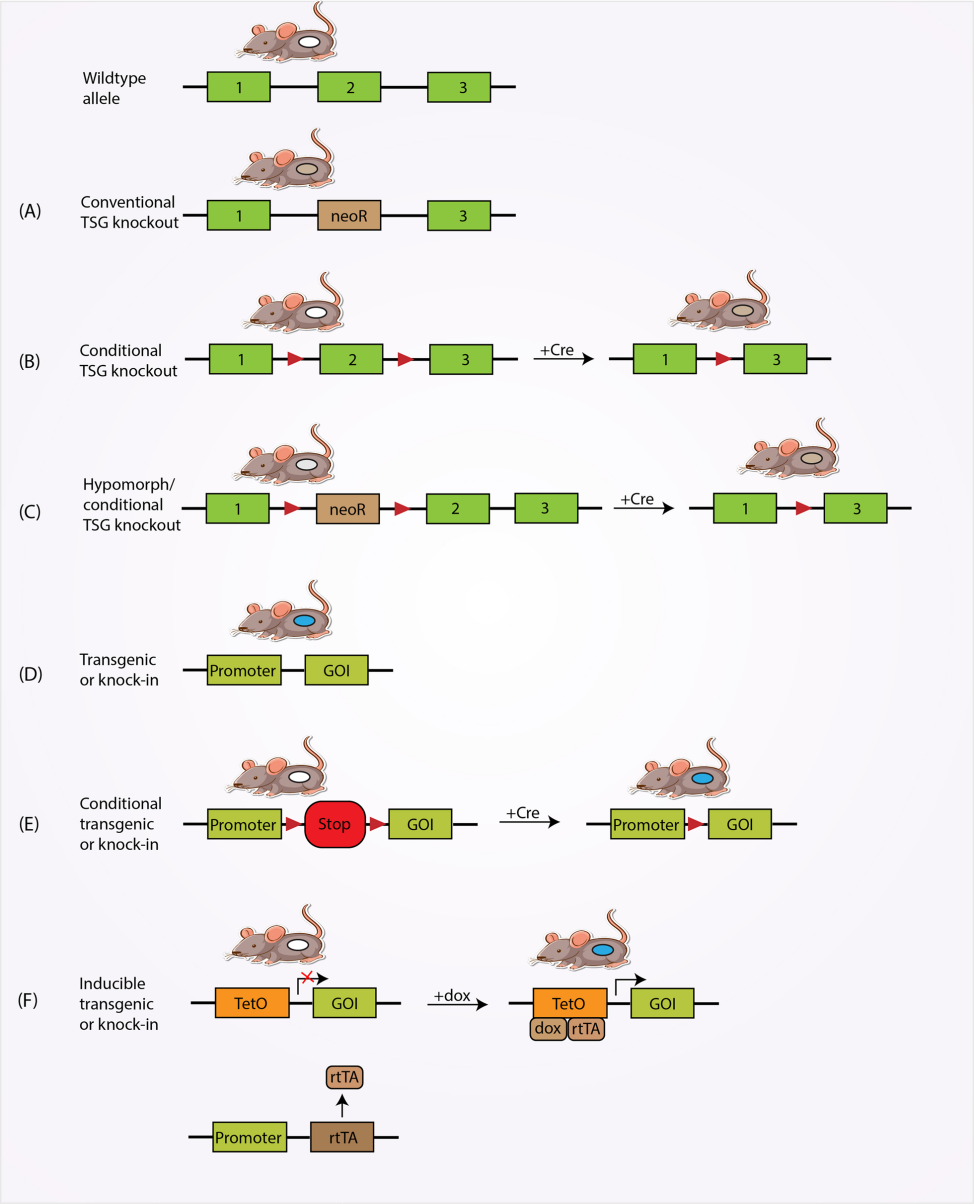
The figure illustrates different types of genetically engineered mouse models (GEMMs) for studying PI3K-driven breast cancer. The color scheme represents different genetic alterations in the mouse tissues. Wildtype tissues are depicted in white, knockout tissues are shown in gray, and tissues expressing a transgene or knock-in gene are represented in blue. **A** Conventional knockout mice exhibit inactivation of tumor suppressor genes (TSGs) in all tissues. **B** Conditional knockout models can be created by flanking critical exons of TSGs with loxP sequences. This allows tissue-specific inactivation of the TSG through Cre site-specific recombinase action, achieved by utilizing a tissue-specific promoter to control Cre expression. **C** Hypomorphic mouse models can be generated by inserting a neomycin cassette into intronic regions, leading to transcriptional interference and reduced expression of the targeted TSG. **D** Constitutive transgenic models involve the insertion of a transgene into the host genome under the control of a tissue-specific promoter (TSP), enabling tissue-specific expression of the gene of interest (GOI). **E** Conditional transgenic systems utilize loxP sites to flank a transcriptional termination sequence (represented by a STOP sign). Recombination by Cre recombinase removes the STOP sequence, allowing tissue-specific expression of the GOI. **F** Doxycycline-inducible 'tet-on' systems rely on the tissue-specific expression of a transactivator (rtTA) that binds to the Tet operator (TetO) and activates expression of the GOI only in the presence of doxycycline (dox). These various GEMMs provide valuable tools to study the role of PI3K signaling in breast cancer by enabling the investigation of tissue-specific effects and the regulation of gene expression

The genetically engineered mouse model (GEMM) has proven to be a valuable preclinical model for evaluating the efficacy of PI3K inhibitors in BC research. These models involve genetically engineering mice to introduce specific mutations in genes associated with BC, such as the PIK3CA gene, which encodes the PI3K catalytic subunit (Fig. 3) [94].

GEMM offers several advantages for the study of PI3K inhibitors in BC (Table 3). By introducing specific genetic alterations, GEMM can closely mimic the molecular properties of human BC, such as activation of the PI3K pathway [94]. This will allow researchers to study the effects of PI3K inhibitors on tumorigenesis, progression and response to therapy in vivo. One of the main strengths of GEMM is its ability to represent the complex nature of BC development and progression. These models mimic the stepwise progression from preinvasive lesions to invasive breast tumors and provide insight into the earliest events of tumorigenesis and the effects of PI3K inhibitors at various stages. GEMM can also be used to assess tumor growth dynamics, metastasis, and interactions between tumors and the surrounding microenvironment [95, 96].

**Table 3:** Advantages and disadvantages of using genetically engineered mouse models as preclinical models

Advantages of genetically engineered mouse models as pre-clinical models	Disadvantages of genetically engineered mouse models as pre-clinical models
Mimicking Molecular Properties of Human BC: GEMM enables the introduction of specific genetic alterations, closely mimicking the molecular properties of human breast cancer, including the activation of the PI3K pathway	Time-Consuming and Expensive: Creating and maintaining GEMM can be time-consuming and expensive, limiting the scalability and efficiency of experiments
Representation of BC Development and Progression: GEMM captures breast cancer's complex development and progression, modeling the stepwise transition from preinvasive lesions to invasive tumors. It provides insights into early tumorigenesis and the effects of PI3K inhibitors at various stages	Limited Reflection of Mutational Landscape: GEMM's introduced genetic alterations may not fully reflect the mutational landscape of human breast cancer, raising concerns about the representativeness of these models
Comprehensive Assessment of Tumor Dynamics: GEMM enables a comprehensive assessment of tumor dynamics, including growth, metastasis, and interactions with the microenvironment, offering a holistic view of breast cancer biology	Integration with Other Models and Clinical Data: To comprehend the effects of PI3K inhibitors in breast cancer, insights from GEMM should be integrated with other preclinical models and clinical data to address potential limitations in genetic fidelity and capture the heterogeneity observed in human tumors
Therapeutic Efficacy and Toxicity Assessment: GEMM allows for the evaluation of therapeutic efficacy and toxicity of PI3K inhibitors by monitoring tumor response, assessing histopathological changes, and analyzing molecular alterations in tumor tissue	
Mechanistic Studies for Drug Response and Resistance: GEMM enables mechanistic studies to understand the molecular mechanisms of response and resistance to PI3K inhibitors. Gene expression manipulation within specific cell types or tumor subtypes helps uncover the role of individual genes or pathways in drug sensitivity or resistance	

GEMM allows assessment of both the therapeutic efficacy and potential toxicity of PI3K inhibitors [97]. Researchers can monitor tumor response to treatment by measuring tumor volume, assessing histopathological changes, and analyzing molecular changes in tumor tissue. In addition, GEMM provides an opportunity to study possible side effects of PI3K inhibitors on normal tissues and organs and helps identify potential off-target side effects.

Furthermore, GEMM enables mechanistic studies to understand the molecular mechanisms underlying response and resistance to PI3K inhibitors [98]. By manipulating gene expression within specific cell types or specific tumor subtypes, researchers can uncover the role of individual genes or pathways in mediating drug sensitivity or resistance [99, 100]. This information can guide the development of combination therapies and the identification of predictive biomarkers for patient stratification.

Despite its advantages, GEMM also has limitations (Table 3). Creating and maintaining them can be time-consuming and expensive. The genetic alterations introduced into GEMM may not fully reflect the mutational landscape found in human BC. Therefore, it is critical to integrate insights from GEMM with other preclinical models and clinical data to understand the effects of PI3K inhibitors in BC fully.

#### 3D organoids

3D organoids have emerged as promising preclinical models for evaluating the efficacy of PI3K inhibitors in BC research [101]. These models are derived from patient-derived cells and self-assemble into three-dimensional structures that mimic the architecture and cellular heterogeneity of the original tumor [102].

There are various advantages of the 3D organoids model (Table 4). One of the major advantages of 3D organoids is their ability to recreate complex tumor microenvironments in a controlled laboratory environment [103]. 3D organoids provide a more physiologically relevant model for assessing the effects of PI3K inhibitors. 3D organoids also preserve patient tumours' genetic and molecular characteristics, such as altered PI3K signaling. This will allow researchers to study the effects of PI3K inhibitors on tumor growth, survival and response to therapy in settings that mimic patient tumors. By using the patient's own cells, 3D organoids offer a personalized approach to drug screening, allowing assessment of individual patient response to PI3K inhibitors.

**Table 4:** Advantages and disadvantages of using 3d organoids as preclinical models

Advantages of 3D Organoids as Pre-clinical Models	Disadvantages of 3D Organoids as Pre-clinical Models
Recreation of Complex Tumor Microenvironments: 3D organoids recreate complex tumor microenvironments, offering a physiologically relevant model for assessing the effects of PI3K inhibitors in a controlled laboratory setting	Incomplete Replication of Tumor Microenvironment Complexity: 3D organoids may not fully replicate the complexity of the tumor microenvironment compared to in vivo models, limiting the assessment of immune responses and drug penetration
Physiologically Relevant Model: 3D organoids preserve genetic and molecular characteristics of patient tumors, including altered PI3K signaling, providing a personalized approach to drug screening and enabling assessment of individual patient responses to PI3K inhibitors	Lack of Immune Cells and Limited Vasculature: The absence of immune cells and limited vasculature in organoids may restrict the evaluation of immune responses and drug penetration. Ongoing efforts to integrate immune cells and vasculature aim to address these limitations
Structural and Functional Similarities to Original Tumor: 3D organoids exhibit structural and functional similarities to the original tumor, allowing the study of important aspects like invasion and metastasis. This provides insights into the effects of PI3K inhibitors on tumor behavior	
High-Throughput Drug Screening: High-throughput drug screening with 3D organoids enables testing of a broad range of PI3K inhibitors and combination therapies simultaneously, offering valuable insights into drug efficacy and identifying candidates for further development	
Long-Term Culture and Monitoring: 3D organoids allow for long-term culture and monitoring of therapeutic response, facilitating the assessment of both short-term and long-term effects of PI3K inhibitors and identification of potential resistance mechanisms	

Furthermore, 3D organoids show structural and functional similarities to the original tumor, enabling the study of important aspects such as invasion and metastasis [104]. These models allow researchers to assess the effects of PI3K inhibitors on the development of tumor invasion and metastatic potential. By incorporating features such as extracellular matrix components, blood vessels, and lymphatics, 3D organoids enable a more comprehensive understanding of the effects of PI3K inhibitors on tumor behavior.

Another advantage of 3D organoids is the potential for high-throughput drug screening [105, 106]. The ability to generate multiple organoids simultaneously allows researchers to quickly and inexpensively test a wide range of PI3K inhibitors and combination therapies. This screening approach provides valuable insight into the efficacy of PI3K inhibitors and helps identify potential drug candidates for further development.

Additionally, 3D organoids offer the potential for long-term culture and monitoring of therapeutic response [107]. Researchers can track changes in organoid growth, morphology, and molecular profile over time, allowing them to assess both short-term and long-term effects of PI3K inhibitors. This longitudinal approach helps identify potential resistance mechanisms and paves the way for developing strategies to overcome drug resistance.

Although 3D organoids have many advantages, they also have some limitations (Table 4). Compared to in vivo models, they may not fully capture the complexity of the tumor microenvironment. The lack of immune cells within the organoids and the limited vasculature may limit the assessment of immune responses and drug penetration. However, efforts are being made to integrate immune cells and vasculature into organoid models to address these limitations.

#### Co-culture models

Co-culture models have emerged as valuable preclinical models for evaluating the efficacy of PI3K inhibitors in BC research. These models utilize co-cultivation of diverse cell types, such as tumor cells and stromal cells, in close proximity to replicate the intricate interactions observed within the tumor microenvironment [108].

Co-culture models have several advantages (Table 5). One of the major advantages of co-culture models is their ability to capture the complexity and heterogeneity of the tumor microenvironment [109]. Breast tumors consist of malignant cells and diverse stromal cell types, including fibroblasts, endothelial cells, and immune cells [110]. The utilization of co-culture models enables the investigation of interactions between tumor and stromal cells, which hold significant importance in both tumor progression and response to therapeutic interventions [111]. These models provide a more physiologically relevant environment for evaluating the effects of PI3K inhibitors.

**Table 5:** Advantages and disadvantages of using co-culture models as preclinical models

Advantages of co-culture models as pre-clinical models	Disadvantages of co-culture models as pre-clinical models
Capture of Tumor Microenvironment Complexity: Co-culture models excel at capturing the complexity and heterogeneity of the tumor microenvironment by incorporating diverse cell types, including tumor and stromal cells, to replicate intricate interactions observed in vivo	Lack of Spatial Organization and 3D Structure: Co-culture models may lack the spatial organization and three-dimensional structure observed in patient tumors, potentially limiting their ability to fully replicate the in vivo environment
Physiologically Relevant Environment: Co-culture models, through the co-cultivation of tumor and stromal cells, offer a more physiologically relevant environment for evaluating the effects of PI3K inhibitors. This reflects the dynamic interactions crucial for tumor progression and response to therapy	Incomplete Replication of Tumor Microenvironment Complexity: While capturing complexity, co-culture models may not fully replicate the intricate and diverse aspects of the tumor microenvironment as observed in vivo
Study of Drug Resistance Mechanisms: Co-culture models facilitate the study of drug resistance mechanisms in the tumor microenvironment, enabling exploration of how stromal cells contribute to resistance. This aids in identifying molecular mechanisms and developing strategies to overcome resistance	Critical Selection of Stromal Cell Types and Ratios: The critical selection of stromal cell types and their ratios in co-culture systems is crucial for model relevance and accuracy. However, this selection process may pose challenges in fully reflecting the diversity of the in vivo tumor microenvironment

Co-culture models also facilitate the study of drug resistance mechanisms with PI3K inhibitors [112]. It is known that the tumor microenvironment may contribute to drug resistance by providing cancer cells with a protective niche. By co-culturing tumor cells with stromal cells, researchers can study the effects of PI3K inhibitors on the development of drug resistance and identify possible molecular mechanisms involved. This information is important for developing strategies to overcome resistance and improve the efficacy of PI3K inhibitors in BC therapy.

Despite its advantages, the co-culture model has limitations (Table 5). They lack the spatial organization and three-dimensional structure observed in patient tumors and may not fully capture the complexity of the tumor microenvironment in vivo. Furthermore, choosing the appropriate stromal cell types and their ratios in the co-culture system is critical to ensure the relevance and accuracy of the model.

## Preventive medicine strategies for breast cancer

Preventive medicine strategies targeting the PI3K pathway in BC may help reduce risks associated with its dysregulated activation.

### PI3K pathway activation

Activation of the PI3K signaling pathway in BC poses a major challenge in prevention and treatment [113, 114], but several preventative medical strategies targeting this pathway show promise [34, 115].

One approach involves the use of PI3K inhibitors [116, 117]. These small-molecule inhibitors specifically block the activity of PI3K, Akt, or mTOR, disrupt downstream signaling cascades, and inhibit tumor growth. Clinical trials have demonstrated the effectiveness of PI3K inhibitor as a treatment for advanced BC [118]. Expanding its use as a prophylactic agent in high-risk individuals or as adjuvant therapy after BC treatment may reduce the risk of cancer recurrence and improve patient outcomes.

Lifestyle changes offer an additional preventive approach. Research indicates that adopting a healthy lifestyle can impact the activation of the PI3K signaling pathway, thereby lowering the risk of BC. Engaging in regular physical activity is associated with a decreased likelihood of developing BC and can positively influence insulin sensitivity [119], and reduce chronic inflammation [120], Both maintaining a healthy weight and consuming a balanced diet rich in fruits, vegetables, and whole grains contribute to reducing the risk of BC. These lifestyle choices potentially affect PI3K signaling, emphasizing their significance in promoting BC prevention [121, 122]. Hormone receptor modulators represent another preventive medicine strategy targeting the PI3K pathway. Endocrine therapies such as selective estrogen receptor modulators (SERMs) and aromatase inhibitors are commonly used for HR+ BC [123, 124]. These agents indirectly affect the PI3K pathway by interfering with estrogen signaling that interacts with the PI3K pathway. Implementing hormone receptor modulators as a preventive intervention in individuals at high risk can substantially decrease the likelihood of developing ER+ BC.

Combination therapies targeting multiple pathways are also expected to prevent PI3K pathway activation in BC. For instance, the synergistic effects and enhanced therapeutic outcomes can be achieved by combining PI3K inhibitors with agents that target other signaling pathways like HER2 inhibitors. The concurrent targeting of both PI3K and HER2 signaling pathways has shown effectiveness in treating HER2-positive BC, highlighting the potential of combination therapy in preventing BC associated with PI3K pathway dysregulation [125].

### Pre-clinical evidence for the preventive effect of PI3K inhibitors

Preclinical evidence proposes that PI3K inhibitors are a promising strategy for preventing BC or arresting disease progression [33, 126]. These inhibitors target the PI3K signaling pathway. The PI3K signaling pathway is a key signaling pathway involved in cell proliferation [127, 128], survival [129, 130], and metabolism [131], which is often dysregulated in BC.

A key finding from preclinical studies is that PI3K inhibitors effectively block the PI3K signaling pathway that is frequently activated in BC [34, 132]. By interfering with this signaling pathway, these inhibitors interfere with cell proliferation and survival, resulting in the suppression of BC growth [117]. Moreover, laboratory models have demonstrated the ability of PI3K inhibitors to suppress the proliferation of BC cells. These inhibitors induce cell cycle arrest, impede cell division, and stimulate apoptosis, thereby preventing tumor progression [133, 134].

Metastasis, the spread of cancer cells to distant organs, is a major challenge in BC treatment. However, preclinical evidence suggests that PI3K inhibitors may help prevent metastasis by targeting key processes involved in cancer cell migration [135], invasion [136], and angiogenesis [137]. By interfering with the PI3K signaling pathway, these inhibitors may impede the metastatic cascade and reduce the likelihood of cancer spreading to other organs.

PI3K inhibitors have also shown the ability to improve the efficacy of other BC treatments. Combining PI3K inhibitors with standard therapies such as chemotherapy [138], and hormone therapy [139], has shown synergistic effects in preclinical studies. This combined approach improves tumor regression and overall survival. This means that PI3K inhibitors may be used as adjunctive therapy to enhance the efficacy of existing treatments and reduce the risk of cancer recurrence.

In addition, preclinical studies have focused on identifying genomic biomarkers that can predict response to PI3K inhibitors [140]. By profiling BC's genomic and molecular features, specific mutations or alterations in genes associated with the PI3K pathway have been identified [141]. These biomarkers may aid in patient stratification and individualized treatment decisions, ensuring that individuals most likely to benefit from PI3K inhibitors are selected. Although preclinical evidence is encouraging, further studies through human clinical trials are important to validate these results. The efficacy, safety and long-term benefits of PI3K inhibitors in BC prevention have yet to be confirmed.

### Mechanisms underlying the preventive effect of PI3K inhibitors in breast cancer

PI3K inhibitors have shown hopeful results as potential preventive agents for BC [142]. To understand the mechanisms underlying the preventive effect, it is important to address the role of the PI3K signaling pathway in BC development and progression.

The PI3K signaling pathway is a key signaling pathway involved in cell growth, survival and metabolism. It is often dysregulated in various cancers, including BC, leading to uncontrolled cell and tumor growth. PI3Ks are crucial in phosphorylating phosphatidylinositol lipids within cell membranes, generating PIP3. Subsequently, PIP3 activates downstream signaling effectors including AKT, mTOR, and other signaling molecules implicated in cell proliferation and survival.

#### Inhibition of PI3K/AKT/mTOR signaling

PI3K inhibitors exert their prophylactic effects by potently blocking PI3K activity, thereby inhibiting the conversion of phosphatidylinositol lipids into PIP3 [143]. This inhibition is vital in decreasing the activation of downstream effectors such as AKT and mTOR [115].

The PI3K/AKT/mTOR signaling pathway helps promote cell survival, growth and angiogenesis [11]. By suppressing this signaling cascade, PI3K inhibitors interfere with key cellular processes such as proliferation and survival that are required for the development and progression of BC.

By inhibiting PI3K, the formation of PIP3, a crucial activator of AKT, is prevented. As a result, reduced AKT activity restricts the activation of downstream targets involved in cell proliferation and survival [144]. Furthermore, inhibition of mTOR, another downstream effector of PI3K, interferes with regulation of protein synthesis and cell cycle progression [145]. By targeting both AKT and mTOR, PI3K inhibitors comprehensively block multiple signaling pathways that drive tumor growth and BC progression.

Overall, inhibition of the PI3K/AKT/mTOR signaling pathway by PI3K inhibitors disrupts a complex network of cellular processes that contribute to BC development. This markedly reduces cell proliferation, promotes apoptosis, inhibits angiogenesis, and ultimately prevents BC tumor formation and progression.

#### Cell cycle arrest and apoptosis induction

An essential mechanism contributing to the protective impact of PI3K inhibitors in BC involves their capability to induce cell cycle arrest [146], and promote apoptosis [147], in BC cells. Dysregulation of the PI3K signaling pathway often disrupts the fine-tuned control of cell cycle progression, allowing cancer cells to evade cell cycle checkpoints and proliferate uncontrollably.

PI3K inhibitors play a central role in restoring proper cell cycle control by targeting key proteins involved in cell cycle progression. By inhibiting the activity of CDKs, central cell cycle regulators, these inhibitors halt cell cycle progression and prevent uncontrolled proliferation of BC cells [148]. PI3K inhibitors suppress tumor growth and prevent BC development by restoring cell cycle checkpoints [149]. In addition, PI3K inhibitors exert a preventive effect by promoting apoptosis, a natural mechanism that eliminates damaged or abnormal cells. Dysregulation of the PI3K signaling pathway often confers anti-apoptotic properties on cancer cells, enabling cancer cell survival and resistance to cell death signals. PI3K inhibitors counter this effect by interfering with anti-apoptotic proteins and promoting the activation of pro-apoptotic signaling pathways [150]. These inhibitors can directly inhibit proteins such as Bcl-2 that inhibit apoptosis, thus shifting the balance towards apoptosis induction in BC cells.

#### Inhibition of angiogenesis

**Fig. 4:**
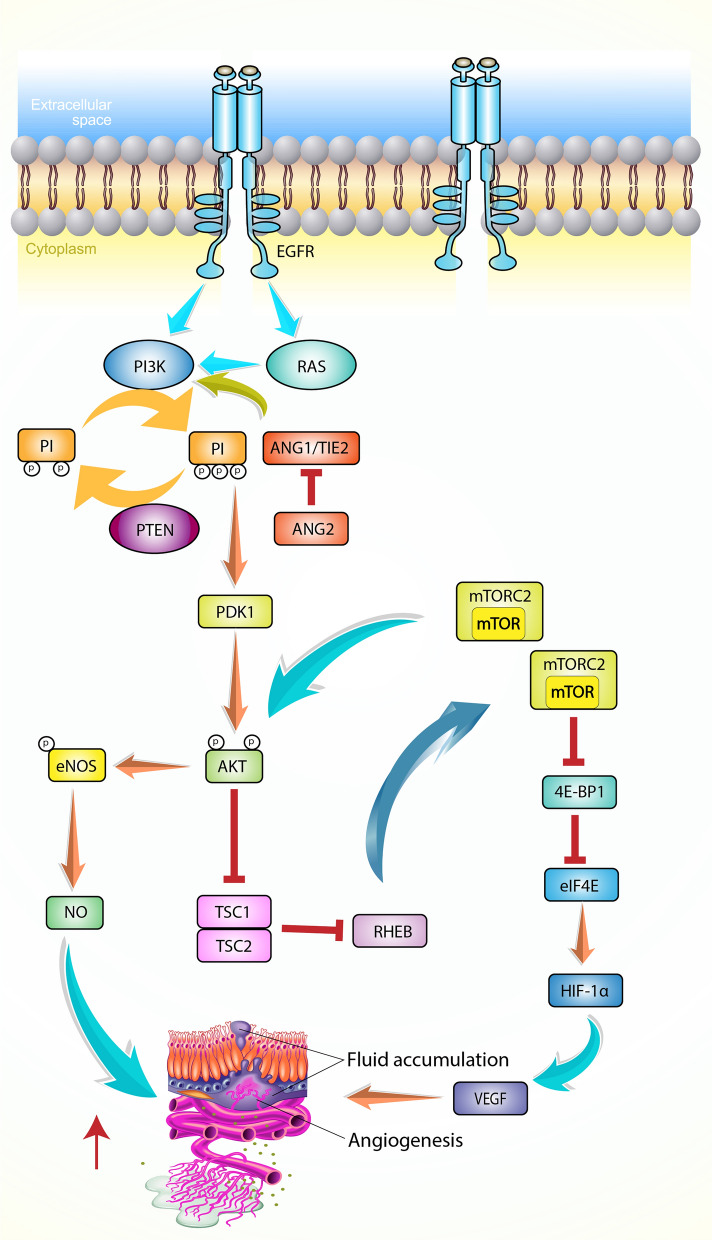
Schematic representation of the PI3K/AKT/mTOR pathway in angiogenesis. Activation of the pathway can occur through multiple mechanisms, including RAS mutation, increased expression of growth factor receptors (e.g., EGFR), or loss of PTEN. Once activated, the pathway leads to the secretion of vascular endothelial growth factor (VEGF), a potent inducer of angiogenesis. Additionally, the PI3K/AKT pathway regulates the expression of other angiogenic factors, such as nitric oxide and angiopoietins

Angiogenesis, the process of forming new blood vessels, is crucial in tumor growth and metastasis. The PI3K signaling pathway assumes a central role in promoting angiogenesis by stimulating the production of pro-angiogenic factors, particularly vascular endothelial growth factor (VEGF) (Fig. 4) [50, 151].

PI3K inhibitors offer a promising approach to prevent angiogenesis in BC [152]. These inhibitors target the dysregulated PI3K signaling pathway and effectively attenuate angiogenesis by inhibiting the expression and secretion of pro-angiogenic factors. In particular, inhibition of PI3K signaling reduces the production of VEGF, a potent inducer of angiogenesis [151]. By inhibiting VEGF and other pro-angiogenic factors, PI3K inhibitors disrupt the delicate balance between stimulators and inhibitors of angiogenesis, thereby inhibiting the formation of new blood vessels required for tumor growth and progression. This decreased angiogenesis limits the blood supply to tumors, deprives them of vital nutrients and oxygen, and ultimately inhibits tumor growth.

Moreover, inhibition of PI3K signaling in endothelial cells lining the inner walls of blood vessels directly affects the cells' ability to form new blood vessels in response to pro-angiogenic signals [153]. PI3K inhibitors disrupt key signaling pathways involved in endothelial cell migration, proliferation, and tubule formation, thereby contributing to the suppression of angiogenesis.

Targeting the PI3K signaling pathway and inhibiting angiogenesis, PI3K inhibitors provide a valuable strategy for preventing the development and progression of BC. By inhibiting pro-angiogenic factors like VEGF, these inhibitors effectively disrupt the formation of new blood vessels, compromising tumor nutrition and impeding tumor growth and metastatic capabilities.

#### Modulation of hormone receptor signaling

Hormone receptor-positive BC is characterized by the presence of estrogen or progesterone receptors on cancer cells, which respond to hormone stimulation and promote tumor growth [154]. The PI3K signaling pathway has been shown to interact with hormone receptor signaling pathways, leading to hormone resistance and contributing to tumor progression in hormone receptor-positive BC [155].

A key mechanism underlying the preventive effects of PI3K inhibitors is disruption of the crosstalk between PI3K signaling and hormone receptor signaling. By targeting the dysregulated PI3K pathway, these inhibitors effectively interfere with communication between the PI3K pathway and hormone receptor signaling, allowing HR+ BC cells to respond to antiestrogenic drugs such as tamoxifen [155].

The PI3K pathway activates downstream effectors such as AKT that directly phosphorylate hormone receptors and alter their activity [143]. This phosphorylation increases hormone receptor activity and may promote resistance to endocrine therapy. PI3K inhibitors counteract this effect by inhibiting AKT activation and downstream signaling, thereby attenuating hormone receptor phosphorylation and activation.

In addition, PI3K inhibitors may modulate other components of hormone receptor signaling pathways. For example, these inhibitors may affect the expression and function of co-regulatory proteins that interact with hormone receptors, thereby affecting hormone receptor activity and response to endocrine therapy.

Combining PI3K inhibitors with endocrine therapy synergistically prevents HR+ BC by targeting multiple signaling pathways, addressing complexity, and improving patient outcomes.

#### Immune modulation

In addition to their direct effects on cancer cells, PI3K inhibitors have shown remarkable immunomodulatory properties by influencing the tumor microenvironment (TME) and enhancing anti-tumor immune responses [156]. The PI3K signaling pathway is vital in suppressing immune cell function and promoting an immunosuppressive environment within tumors [157].

PI3K inhibitors counteract these immunosuppressive effects by promoting immune cell activity and promoting immunostimulatory TME. One of the key mechanisms by which PI3K inhibitors exert immunomodulation is by enhancing the function of immune cells such as T cells and natural killer (NK) cells [158]. Activation of the PI3K signaling pathway in immune cells can impair the cytotoxic capacity of immune cells and weaken anti-tumor responses. By inhibiting PI3K, these inhibitors restore immune cell function and enhance the ability to recognize and eliminate cancer cells.

Furthermore, PI3K inhibitors modulate the TME and promote an immunostimulatory environment by hindering the recruitment and activation of immunosuppressive cells like regulatory T cells (Treg) [159]. Furthermore, PI3K inhibitors can reduce the production of immunosuppressive factors such as cytokines and chemokines while promoting the secretion of immunostimulatory factors [160].

The immunomodulation exerted by PI3K inhibitors helps prevent tumor formation and progression. By enhancing immune cell activity and promoting an immune-stimulating TME, these inhibitors facilitate the immune system's recognition and elimination of cancer cells, thereby hindering tumor growth and metastasis.

#### Interplay between metabolism and PI3K inhibitors

The intricate interplay between metabolism and PI3K inhibitors in breast cancer underscores the nuanced nature of targeted therapy. Metabolically, breast cancer cells prominently exhibit the Warburg effect, marked by heightened glucose uptake and glycolysis to sustain rapid cellular proliferation [161, 162]. Altered lipid metabolism and increased reliance on specific amino acids further accentuate the metabolic demands associated with abnormal growth.

PI3K inhibitors specifically intervene in this metabolic milieu by targeting the dysregulated PI3K signaling pathway, a common feature in breast cancer progression [163]. Mutations in the PIK3CA gene contribute to the hyperactivation of PI3K signaling, underscoring the therapeutic significance of PI3K inhibitors [164, 165]. Designed to selectively target isoforms such as p110α, PI3K inhibitors disrupt downstream signaling events involving molecules like AKT and mTOR. This targeted intervention disrupts the orchestrated processes that maintain cancer cell viability and uncontrolled growth [163].

This precision in targeting aligns with the metabolic vulnerabilities of breast cancer cells, presenting a strategic approach to disrupt signaling cascades intricately linked to cellular metabolism [166]. Clinical trials evaluating PI3K inhibitors, such as alpelisib and taselisib, have demonstrated their potential in breast cancer treatment. These studies explore the synergistic effects of PI3K inhibitors in combination therapy, often integrating them with endocrine therapy, HER2-targeted therapy, or chemotherapy to maximize therapeutic efficacy [34, 167].

Patient stratification based on molecular profiling becomes paramount to optimize the prophylactic efficacy of PI3K inhibitors [168]. Identifying PIK3CA mutations enables a personalized approach, ensuring that individuals most likely to benefit from PI3K inhibitor therapy are precisely targeted [169, 170]. This tailored strategy not only optimizes treatment outcomes but also minimizes potential side effects [171, 172].

Despite these advancements, challenges persist, including the emergence of resistance to PI3K inhibitors and the intricate regulatory mechanisms within the PI3K signaling pathway [173]. Ongoing research efforts aim to unravel this complexity, refining preventive strategies and providing insights to further advance breast cancer treatments [6, 174]. The dynamic interactions between metabolism and PI3K inhibitors offer a promising avenue for the development of more effective and personalized therapies in the context of breast cancer [175].

## Personalized medicine approaches for breast cancer

Personalized medicine approaches in BC focus on identifying factors that influence treatment response, particularly in relation to the PI3K pathway. Several key factors play a role in determining the efficacy of PI3K inhibitors and can guide personalized treatment decisions:

### Tumor stage

Breast cancer stage is an important factor in selecting the most suitable treatment strategy [176]. This includes various aspects such as tumor size, nearby lymph node involvement, and distant metastases. These factors guide treatment decisions and help determine each patient's best course of action.

In early-stage BC where the tumor is localized and has not spread beyond the breast or nearby lymph nodes, may benefit from the use of PI3K inhibitors as part of adjuvant therapy [177]. Adjuvant therapy refers to additional treatment administered after initial procedures like surgery or radiation to reduce the risk of cancer recurrence [178]. The goal is to target BC's dysregulated PI3K signaling pathways by including PI3K inhibitors in adjuvant therapy regimens. This targeted approach prevents cancer cells from growing and reduces the risk of recurrence.

In patients with advanced or metastatic BC whose cancer has spread to other parts of the body, PI3K inhibitors are often part of systemic treatment [85, 179]. Systemic treatment aims to manage cancer growth and metastasis throughout the body effectively. In this regard, PI3K inhibitors target the aberrant HER/PI3K signaling pathway, significantly influencing cancer cell proliferation and survival [180]. Blocking this signaling pathway has the potential to slow or halt disease progression, alleviate symptoms, and improve overall survival.

It should be noted that treatment decisions are made individually, considering factors such as the patient's general health status, genetic profile, specific BC subtype, and other relevant clinical factors. Therefore, the use of PI3K inhibitors may vary depending on the patient's unique characteristics and the stage of BC. Close collaboration between the patient and the medical team is essential to determine the most appropriate treatment plan for optimal results.

### Molecular subtypes

Breast cancer has several molecular subtypes, including but not limited to HR+ BC [181, 182], HER2+ BC [183, 184], and triple-negative breast cancer (TNBC) [185, 186]. Classifying BC into molecular subtypes is essential as it provides important insights into tumor biology and helps in adjusting therapeutic approaches accordingly [187, 188].

One of the most important applications of molecular subtyping in the treatment of BC is the selection of targeted therapies [189]. PI3K inhibitors have exhibited effectiveness when combined with other targeted therapies against specific molecular subtypes. Notably, cases of HR+ BC with mutations in the PIK3CA gene, responsible for encoding the PI3K enzyme, have displayed distinct responses to PI3K inhibitors [190]. HR+ BC refers to tumors that express estrogen and/or progesterone receptors. This subtype often benefits from endocrine therapies such as hormone receptor blockers and aromatase inhibitors that interfere with hormone signaling pathways. However, in HR+ BC cases with PIK3CA mutations, dysregulated PI3K signaling may contribute to resistance to endocrine therapy [191]. Adding a PI3K inhibitor to the treatment regimen can overcome this resistance and improve treatment outcomes.

It is crucial to acknowledge that PI3K inhibitors and other targeted therapies are often tailored to specific molecular subtypes of BC due to variations in efficacy and applicability. Identifying PIK3CA mutations through molecular profiling or genetic testing can aid in selecting patients who are more likely to benefit from PI3K inhibitors as part of their treatment.

### Genetic alterations

Genetic changes within the PI3K signaling pathway, particularly PIK3CA gene mutations, significantly influence the response to therapy in BC [192, 193]. Such alterations can affect the activation or dysregulation of PI3K signaling pathways, which play a vital role in cell growth, survival and proliferation.

BC patients with PIK3CA mutations show increased sensitivity to PI3K inhibitors, making them potential candidates for targeted therapy [194]. Genetic testing plays an important role in identifying patients who are likely to respond positively to PI3K inhibitor therapy. Analyzing the tumor's genetic profile, particularly PIK3CA mutations, will allow healthcare providers to individualize treatment decisions and select the most appropriate treatment option [195]. Identifying patients with PIK3CA mutations will enable targeted therapeutic approaches that specifically administer PI3K inhibitors to block signaling defects in the PI3K pathway [195]. This targeted therapy aims to block the activity of the PI3K enzyme, whose activity is increased by genetic alterations, thereby inhibiting the growth and survival of cancer cells.

Genetic testing is typically performed using various methods such as DNA sequencing technology to detect specific mutations within the PIK3CA gene [196]. It is essential to recognize that PIK3CA mutations are absent in all BC patients. Hence, genetic testing is crucial in identifying specific subsets of patients who are most likely to derive significant benefits from PI3K inhibitor therapy.

Incorporating genetic testing into the clinical setting will enable healthcare providers to optimize treatment decisions and deliver precision medicine to BC patients. This approach maximizes the likelihood of a positive treatment response while minimizing unnecessary treatment exposure for individuals unlikely to benefit from PI3K inhibitors.

### Biomarker analysis

A comprehensive analysis of biomarkers, including genomic profiling, is of great value in understanding BC treatment outcomes [47, 197]. In addition to PIK3CA mutations, alterations in other genes within PI3K pathway, such as PTEN [198, 199], serve as important biomarkers for patient stratification and prediction of response to PI3K inhibitors.

Genomic profiling allows us to study the genetic makeup of tumors in detail and identify specific alterations and abnormalities within the PI3K signaling pathway. Besides PIK3CA mutations, biomarker analyses include assessing PTEN, a tumor suppressor gene, and AKT, a downstream effector of PI3K signaling [200]. These biomarkers may provide important insights into the dysregulation of PI3K signaling pathways and influence therapeutic decisions. Integrating biomarker analysis, including genomic profiling, into clinical trial design enables a more individualized and customized therapeutic approach. By identifying patients with specific biomarker profiles, such as changes in PIK3CA, PTEN and AKT, researchers can select participants who are likely to respond positively to PI3K inhibitor treatment [201]. This approach will improve the efficacy and efficiency of clinical trials and provide a better understanding of treatment response within specific patient subgroups.

Furthermore, biomarker analysis helps in patient stratification and offers the potential for personalized treatment approaches [202, 203]. By considering each patient's unique biomarker profile, healthcare providers can optimize treatment decisions and select the most appropriate therapeutic agents, such as PI3K inhibitors that target specific molecular alterations within tumors.

### Host factors

Host factors, including individual patient characteristics, greatly influence response to BC therapy [204]. Factors such as age [205], general health status [206], and genetic diversity can significantly influence various aspects of treatment, such as drug metabolism, drug interactions, and tolerability [207]. Recognizing these host factors and incorporating them into treatment decisions allows for a more individualized and customized treatment approach that addresses each patient's unique needs.

Age plays an important role in considering treatment. Older patients may have differences in drug metabolism and tolerability compared to younger individuals [208, 209]. Additionally, additional comorbidities and age-related factors may necessitate treatment plan adjustment. Conversely, younger patients may have other fertility-sparing concerns and long-term side-effect considerations that need to be addressed [210].

The patient's overall health status, including organ function, performance status, and presence of comorbidities, is important in determining treatment options and dose adjustments. Associated medical conditions may affect a patient's ability to tolerate a particular therapy or influence treatment outcome [211]. For example, patients with hepatic or renal impairment may require dose adjustments or alternative therapeutic approaches to ensure optimal efficacy and safety. Genetic differences between individuals can significantly impact therapeutic response and drug metabolism. Polymorphisms in genes involved in drug pathways can affect drug efficacy and toxicity. Genetic testing can help identify specific mutations that may affect metabolism or response to certain treatments, and aid in individualized treatment decisions [212].

Considering these host factors allows healthcare providers to tailor treatment to individual patient needs. Tailoring treatment to address age-related considerations, general health conditions and genetic variability to ensure optimal treatment outcomes while minimizing potential risks and side effects. Incorporating these factors into treatment decisions promotes a patient-centred approach that considers each individual's unique characteristics and circumstances.

## Challenges in developing PI3K inhibitors for breast cancer

In BC, PI3K inhibitors exhibit a distinct toxicity and side effect profile. The management strategies for mitigating the toxicity of PI3K inhibitors are discussed below.

### Toxicity

Although toxicity has been a major obstacle in developing PI3K inhibitors for the treatment of BC [180], the PI3K signaling pathway plays a vital role in multiple cellular processes related to cell proliferation, survival, and metabolism, and may be therapeutically useful.

A targeted approach is needed to address the toxicity challenge, especially in BC therapy [183, 213]. The goal is to develop PI3K inhibitors with a better therapeutic window that allows selective targeting of cancer cells with minimal toxicity to normal breast tissue. To achieve this, researchers are investigating strategies to increase the selectivity of PI3K inhibitors for BC cells. This includes the development of inhibitors that specifically target isoforms of PI3K that are often dysregulated in BC, such as PI3Kα [214, 215], and PI3Kβ [216]. Targeting these isoforms can reduce off-target side effects and minimize toxicity to normal cells.

Additionally, researchers are exploring new drug delivery systems to improve the accumulation and retention of PI3K inhibitors in BC cells [217]. Targeted delivery mechanisms such as nanoparticle-based systems [218, 219] and antibody–drug conjugates [220, 221] can be used to increase the concentration of inhibitors within tumor cells while sparing healthy breast tissue. This approach helps to reduce the potential for toxicity further.

### Isoform-specificity

Isoform specificity poses a major challenge in the development of PI3K inhibitors for the treatment BC [222]. PI3K is composed of four distinct isoforms (α, β, γ, δ), each with unique roles in cellular processes [223]. Various subtypes of BC may depend on specific isoforms of PI3K in their proliferation and survival. Therefore, the development of inhibitors that selectively target relevant isoforms involved in BC while sparing other isoforms is an important goal.

Breast cancer subtypes, including HR+, HER2+, and TNBC, show different dependencies on PI3K isoforms. For example, HR+ BC often show dysregulated activation of the PI3Kα isoform [224], which is associated with resistance to hormone therapy [191]. In contrast, activation of both PI3Kα and PI3Kβ isoforms is common in HER2+ BC [225].

To address this challenge, researchers are focusing on developing isoform-specific PI3K inhibitors that selectively target isoforms involved in BC subtypes [226]. Understanding the specific isoform dependencies of different BC subtypes has allowed scientists to develop isoform-targeted inhibitors that promote cancer growth while sparing less relevant isoforms [227].

Two approaches are being pursued to selectively inhibit PI3K isoforms. The first approach involves rational design of inhibitors with structural modifications that exploit the inherent differences between isoforms. This enables the development of isoform-specific inhibitors, reducing off-target effects and toxicity. The second strategy involves computational methods and virtual screening to identify isoform-specific PI3K inhibitors. Researchers can screen chemical libraries for compounds with high affinity and selectivity using computer models and molecular docking, optimizing isoform specificity and potency. Additionally, combination therapies targeting multiple isoforms simultaneously or sequentially are being explored to improve efficacy while considering the complex heterogeneity and overlapping functions of PI3K isoforms in BC.

### Isoform-specific inhibitors

**Fig. 5:**
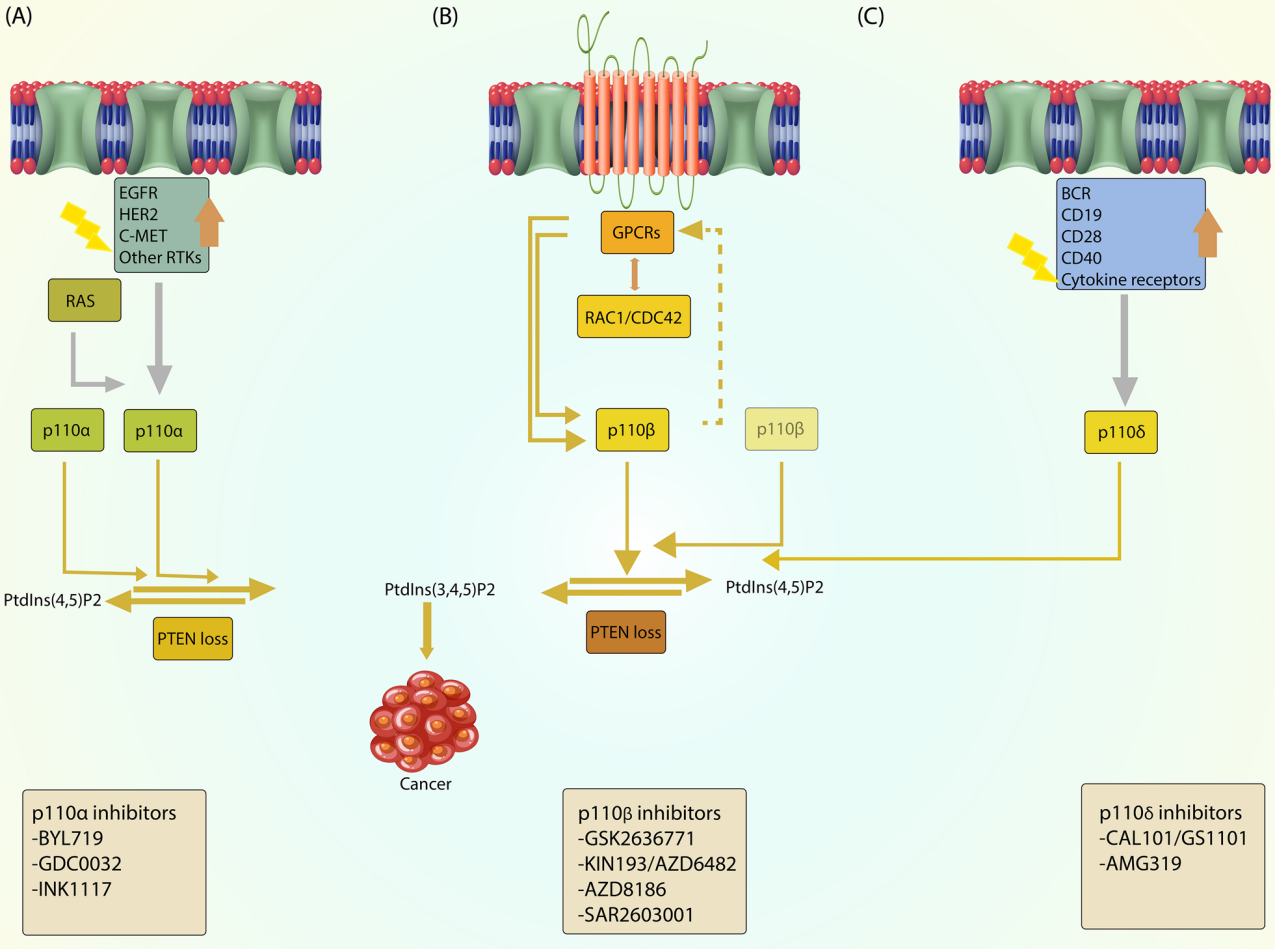
The figure illustrates the molecular contexts that determine the applications of isoform-selective PI3K inhibitors. It represents different molecular alterations and their effects on PtdIns(3,4,5)P3 production through specific PI3K isoforms. **A** Upregulation or mutation of receptor tyrosine kinases (RTKs), oncogenic RAS mutations, or activating p110α mutations result in increased PtdIns(3,4,5)P3 production through p110α, which can be further amplified by mutation or loss of PTEN. In these contexts, the use of p110α-selective inhibitors is effective. **B** In the absence of other oncogenic alterations, loss or mutation of PTEN increases PtdIns(3,4,5)P3 production through p110β, potentially due to RAC1- or CDC42-mediated p110β activation or the basal activity of this isoform. In this context, the use of p110β-selective inhibitors is effective. **C** Upregulation or mutation of B cell receptors (BCRs), cytokine receptors, or other immune cell surface markers leads to increased PtdIns(3,4,5)P3 production through p110δ. In this context, the use of p110δ-selective inhibitors is effective. These different molecular alterations and their specific effects on PI3K isoforms highlight the rationale for using isoform-selective PI3K inhibitors based on the molecular context of the disease. This knowledge can guide the development and application of targeted therapies for various contexts associated with PI3K pathway dysregulation

Recent advances in drug discovery have led to the development of isoform-specific PI3K inhibitors, offering promising opportunities for treating BC (Fig. 5). By targeting isoforms specifically linked to certain BC subtypes, these inhibitors have the potential to enhance treatment effectiveness while reducing adverse effects [228, 229].

The subtypes exhibit different characteristics and treatment responses, necessitating customized therapeutic approaches. For instance, dysregulated PI3Kα isoform signaling is often observed in HR+ BC. By developing isoform-specific inhibitors that selectively target PI3Kα, aberrant signaling pathways promoting proliferation and survival in HR+ BC cells can be more effectively inhibited while minimizing toxicity to normal cells [230, 231].

Furthermore, in TNBC, PI3Kγ isoforms were found to play central roles in tumor growth and progression. The development of isoform-specific inhibitors that selectively target these isoforms would provide a targeted approach to disrupt PI3K signaling, particularly in TNBC cells [232], potentially overcoming resistance mechanisms and reducing this malignancy. It may improve treatment response in high-grade BC subtypes. Advances in structural biology, computational modeling, and medicinal chemistry techniques have enabled researchers to rationally design isoform-specific PI3K inhibitors. By exploiting each isoform's unique structural features and binding pockets, it becomes possible to design inhibitors that selectively bind and inhibit target isoforms while sparing other isoforms. This approach increases inhibitor selectivity, reduces off-target effects, and improves therapeutic index.

### Biomarkers and patient stratification

The Reliable biomarkers are crucial in BC therapy with PI3K inhibitors. They aid in patient stratification, identifying those who will likely respond well to treatment. Biomarkers enable personalized approaches, improving the chances of successful outcomes [140, 233]. Additionally, the discovery of predictive biomarkers will aid in monitoring therapeutic response and early detection of resistance among BC patients. This enables more precise and timely interventions to optimize treatment outcomes.

Identifying biomarkers associated with susceptibility to PI3K inhibitors, such as mutations in the PIK3CA gene, can optimize treatment outcomes in BC. PIK3CA mutations are prevalent in BC and serve as a useful biomarker for guiding treatment decisions [234], especially in HR+ and HER2 negative subtypes [235]. PIK3CA mutations increase sensitivity to PI3K inhibitors, helping identify patients for targeted therapy. Other gene alterations in the PI3K pathway, like PTEN and AKT, can also serve as biomarkers for response prediction. Loss of PTEN function or AKT activation may cause resistance to PI3K inhibitors [236].

Advances in genomic profiling techniques such as next-generation sequencing and gene expression profiling have enabled the identification of additional biomarkers associated with PI3K inhibitor response in BC. By analyzing the molecular properties of tumors, specific genomic alterations, gene expression patterns, or activation signatures can be identified as potential predictive biomarkers [237, 238]. These biomarkers help classify patients into responders and non-responders, allowing for more individualized therapeutic approaches and improved clinical outcomes.

Additionally, biomarkers may play a role in monitoring treatment response and detecting early signs of resistance. Continuous monitoring of biomarkers during treatment can provide insight into the efficacy of PI3K inhibitors and detect potential resistance mechanisms that may emerge. Alterations in biomarker profiles can indicate response to therapy, disease progression, or the development of resistance, allowing timely adjustments in therapeutic strategies and exploration of alternative therapeutic options.

### Preclinical and clinical trial design

Improving preclinical models and optimizing clinical study designs are essential to gain valuable insight into the toxicity and side effect profiles of PI3K inhibitors in BC. These efforts will contribute to the development of more effective drugs and help identify strategies to reduce toxicity associated with PI3K inhibitors.

Preclinical models, like cell cultures and animal models, assess PI3K inhibitor efficacy and safety before human trials [85]. Enhancing breast cancer-specific preclinical models improves accuracy in predicting response. PDX models transplant BC tissue into mice, enabling evaluation in a clinically relevant context. PDX models uncover molecular subtypes and genetic alterations that influence PI3K inhibitor sensitivity or resistance, aiding targeted therapy development.

In addition to preclinical models, it is important to optimize clinical trial designs to assess the safety and efficacy of PI3K inhibitors in BC patients [42, 116]. Well-controlled clinical trial design, including appropriate patient stratification, outcome measures, and monitoring protocols, is essential to obtain reliable data on drug toxicity profiles. This includes considering factors such as BC subtype, hormone receptor status, and the presence of certain genetic alterations (such as PIK3CA mutations) to guide patient selection and assessment of treatment response. Clinical trials rigorously assess and monitor the toxicity of PI3K inhibitors in BC patients. Comprehensive evaluation and reporting of adverse events enable identifying common toxicities and the development of mitigation strategies. This enhances patient tolerability and treatment adherence. Integrating biomarker analysis in clinical trials enhances patient selection, treatment response prediction, and toxicity identification. Molecular profiling identifies drug susceptibility, resistance, and toxicity biomarkers, enabling personalized therapies. This optimizes outcomes by targeting patient subsets likely to benefit from PI3K inhibitors.

### Resistance mechanisms to PI3K inhibitors

Resistance to PI3K inhibitors can arise through various mechanisms, limiting their long-term effectiveness in cancer treatment. Some common resistance mechanisms include:

#### Activation of alternative signaling pathways

**Fig. 6:**
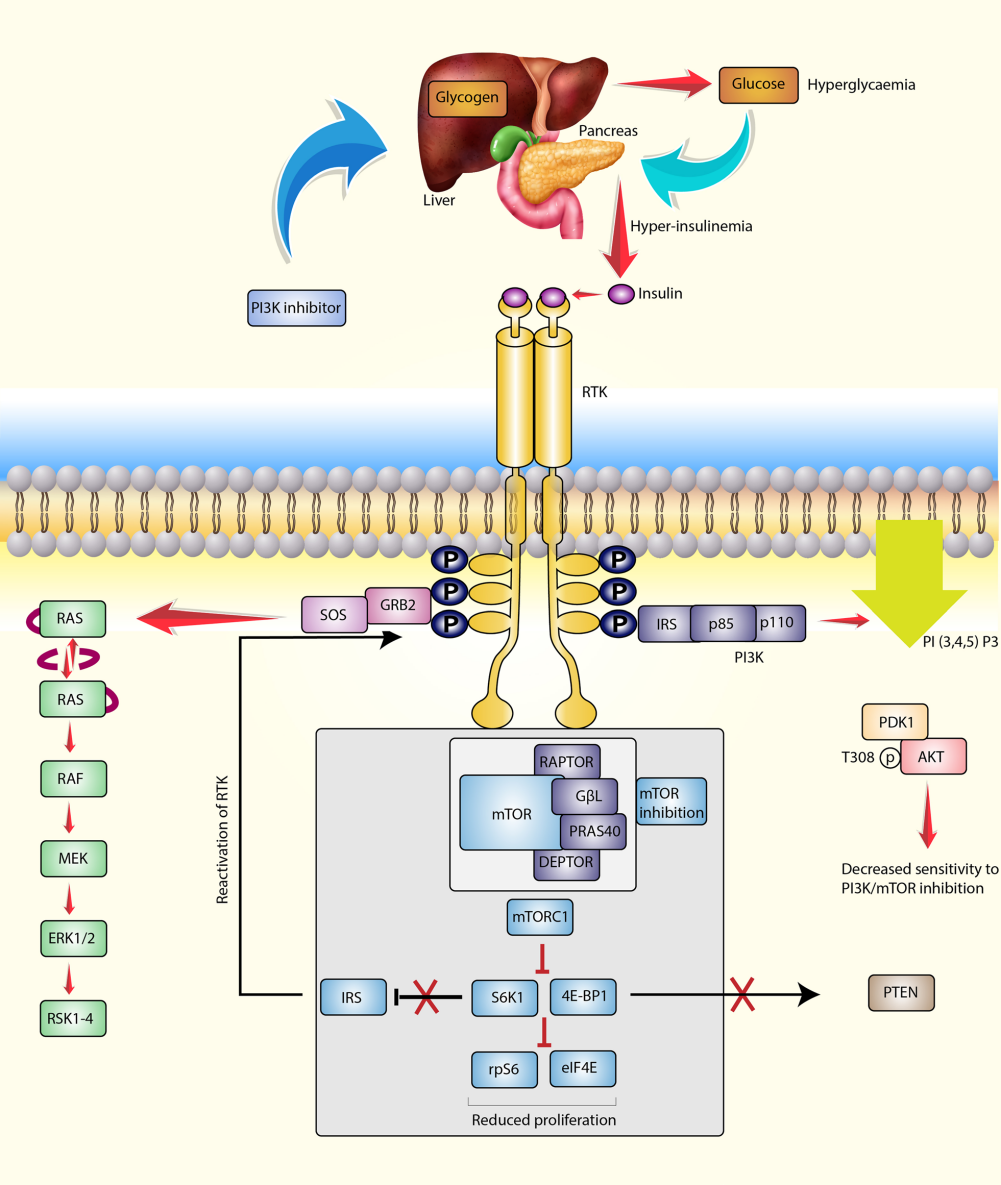
Insulin-Mediated Feedback Loops Following PI3K/mTOR Inhibition: This figure illustrates the complex feedback loops that occur following treatment with PI3K and mTOR inhibitors in cancer cells. When PI3K is inhibited, the liver breaks down stored glycogen, releasing glucose into the bloodstream, resulting in hyperglycemia. The pancreas detects elevated glucose levels, triggering a substantial insulin release, leading to hyperinsulinemia. Hyperinsulinemia partially reactivates the insulin receptor, thereby reinstating the activity of insulin receptor substrate (IRS) and growth factor receptor-bound protein 2 (GRB2). This reactivation leads to an increase in both PI3K and MAPK pathway activation, limiting the therapeutic effects of PI3K inhibitors. Similarly, mTOR1 inhibitors, such as rapamycin, downregulate S6 kinase 1 (S6K1) and eukaryotic translation initiation factor 4E-binding protein 1 (4E-BP1), which blocks downstream translation. The de-repression of 4E-BP1 leads to the enhanced availability of IRS1, acting as an intermediary between the insulin receptor and the PI3K complex. This recruitment of PI3K to the active receptor enhances both MAPK and downstream PI3K signaling, thereby reducing the overall sensitivity to mTOR inhibition. Downregulation of mTOR activity through AKT inhibition or direct mTOR inhibition blocks 4E-BP1-mediated phosphatase and tensin homolog (PTEN) translation. This results in the accumulation of phosphatidylinositol 3,4,5-trisphosphate (PIP3), leading to sustained activation of AKT

Cancer cells show remarkable adaptability to PI3K inhibition, which can activate alternative signaling pathways and circumvent the silencing effects of PI3K inhibitors. A key mechanism in these cells is the activation of parallel signaling pathways such as the MAPK pathway (mitogen-activated protein kinase) and the mTOR pathway. Cancer cells can evade the blocking of PI3K signaling by using these signalling pathways, thus ensuring cancer cell survival and uncontrolled proliferation (Fig. 6) [9].

The MAPK signaling pathway is pivotal in governing fundamental cellular processes, including cell proliferation and division [239, 240]. In response to PI3K inhibition, cancer cells may activate the MAPK pathway as an alternative way to sustain proliferation. Through this activation, cancer cells can evade the effects of PI3K inhibitors, facilitating their survival and proliferation despite the presence of these targeted therapies.

The mTOR pathway, a critical regulator of cell proliferation and protein synthesis, acts as a compensatory pathway when PI3K is inhibited. Cancer cells can upregulate mTOR pathway activity, even in the presence of PI3K inhibitors. This allows them to sustain growth and proliferation despite the blockade of PI3K signaling [241].

By activating these parallel signaling pathways, cancer cells evade the target's inhibition of PI3K, thereby conferring resistance to PI3K inhibitors. This adaptive response highlights the complex nature of cancer and the challenges in developing effective therapeutic strategies against dysregulated PI3K signaling.

#### Mutations in the PI3K pathway

Mutations in the PI3K signaling pathway pose a significant challenge to the efficacy of PI3K inhibitors in cancer therapy, particularly secondary to key components such as the PIK3CA gene and other essential components of the signaling pathway [194]. Mutations can impair the efficacy of PI3K inhibitors. These mutations can cause structural or functional alterations in PI3K or its downstream targets, thereby reducing the efficacy of inhibitors that block signaling pathways. As a result, cancer cells harboring such mutations retain the ability to maintain dysregulated activation of the PI3K pathway, ultimately resulting in resistance to PI3K-targeted therapy [242].

Specifically, mutations in the PIK3CA gene, responsible for encoding the PI3K catalytic subunit, can lead to the continuous activation of PI3K signaling, rendering it unaffected by inhibitors [243]. These mutations often involve changes in key domains of the protein, such as the kinase domain, resulting in increased enzymatic activity and maintenance of downstream signaling. Furthermore, mutations in other components of the PI3K signaling pathway, including PTEN, impair negative feedback regulation and enhance activation of signaling pathways, leading to resistance [244].

#### Upregulation of compensatory pathways

The adaptability of cancer cells to PI3K inhibition is demonstrated by their ability to upregulate compensatory pathways such as the MAPK and mTOR pathways [126]. These alternative pathways act as countermeasures against PI3K blockade, allowing cancer cells to maintain proliferation despite inhibitory effects.

Compensatory upregulation of the MAPK signaling pathway is common in cancer cells in the presence of PI3K inhibitors [245]. The MAPK signaling pathway plays a pivotal role in governing cellular processes including cell growth, proliferation, and survival. Cancer cells can exploit this pathway by amplifying its activity, bypassing the inhibitory effects of PI3K inhibitors and promoting unchecked cancer cell proliferation. This activation ensures that critical cellular functions required for survival and proliferation are maintained even in the presence of PI3K inhibitors.

Similarly, cancer cells use upregulation strategies involving the mTOR signaling pathway as compensatory mechanisms. The mTOR pathway is a key regulator of protein synthesis, cell growth and proliferation [246]. PI3K inhibition causes cancer cells to increase the activity of mTOR pathway to compensate for loss of PI3K signaling. This upregulation provides an alternative way to sustain PI3K proliferation and overcome the inhibitory effects of PI3K inhibitors. By activating these compensatory pathways, cancer cells establish mechanisms to overcome blockade of PI3K signaling and ensure survival and proliferation. Upregulation of the MAPK and mTOR signaling pathways allows cancer cells to compensate for PI3K loss of function and maintain proliferative potential despite treatment with PI3K inhibitors.

#### Loss of negative regulators

Loss of PTEN, a key negative regulator of PI3K signaling [247], plays an important role in conferring resistance to PI3K inhibitors [248]. PTEN normally functions as a tumor suppressor by inhibiting PI3K signaling [249]. However, when PTEN is lost or inactivated by genetic alterations or other mechanisms, the PI3K pathway is overactivated. This loss of PTEN function results in dysregulation of the PI3K signaling pathway, resulting in increased cell proliferation and survival signals.

When cancer cells lose functional PTEN, they become less susceptible to the inhibitory effects of PI3K inhibitors [58]. In the absence of PTEN, the PI3K pathway is persistently misactivated, evading the intended therapeutic effects of PI3K inhibitors. As a result, cancer cells lacking functional PTEN may continue to proliferate uncontrolled and evade the inhibitory effects of PI3K inhibitors.

Loss or inactivation of PTEN can occur through various mechanisms, including genetic mutations [250], epigenetic modifications [251], and altered protein stability [252]. These alterations disrupt normal PTEN function and cause imbalances within the PI3K signaling pathway.

Understanding the importance of PTEN loss in the emergence of resistance to PI3K inhibitors is critical for the development of effective therapeutic strategies. Therapeutic approaches targeting restoration of PTEN function and alternative vulnerabilities in PTEN-deficient cancer cells may overcome resistance and improve outcomes of PI3K-targeted therapies.

## Opportunities in developing PI3K inhibitors for breast cancer

Developing PI3K inhibitors for BC presents promising opportunities to enhance treatment strategies and improve patient outcomes. These opportunities include:

### Overcoming resistance to PI3K inhibitors

The development of resistance to PI3K inhibitors is a major challenge in BC therapy. Strategies to overcome resistance to PI3K inhibitors are described below.

#### Dual inhibition

A dual inhibition strategy has emerged as a powerful approach to improve the efficacy of PI3K-targeted therapy by blocking compensatory signals and overcoming resistance [253]. These strategies simultaneously target multiple components of the PI3K pathway to achieve comprehensive and synergistic blockade, ultimately improving therapeutic outcomes.

The PI3K signaling pathway consists of several key components, including PI3K itself, downstream effectors such as AKT and mTOR [145]. Cancer cells often evolve compensatory mechanisms by activating alternative signaling pathways when a single component of the PI3K signaling pathway is attacked. To circumvent this resistance, dual-inhibition approaches aim to simultaneously target multiple components of the PI3K signaling pathway. One strategy involves combined inhibition of PI3K and its downstream effector mTOR. This dual-inhibition approach effectively suppresses two key nodes in the signaling pathway, resulting in broader blockade. Simultaneous targeting of PI3K and mTOR prevents compensatory signaling through mTOR activation, enhances therapeutic efficacy, and overcomes resistance to PI3K inhibitors [33, 115].

A dual inhibition strategy targeting both PI3K and AKT maybe effective in disrupting the signaling cascade and preventing compensatory activation of AKT, leading to potent inhibition of downstream events. Combining PI3K inhibitors with agents targeting alternative pathways, such as the MAPK pathway, may further enhances therapeutic response by preventing compensatory activation of MAPK signaling and suppressing pro-survival signals. This comprehensive approach will hold promise for overcoming resistance mechanisms and improving outcomes in PI3K-associated cancers.

#### Novel agents targeting resistance mechanisms

The development of new drugs that specifically target and overcome known resistance mechanisms is an important tool for improving cancer outcomes [254]. Identifying and targeting specific resistance mechanisms that emerge during therapy. By doing so, these new drugs have the potential to be transformative in order to increase the efficacy of targeted therapies [255].

Resistance mechanisms occur through various processes such as genetic alterations, epigenetic modifications, and activation of compensatory pathways. Understanding the specific mechanisms of resistance allows researchers to design drugs that directly target and counteract these mechanisms [255].

For example, in the context of therapies targeting the PI3K signaling pathway, new drugs may be developed that selectively block secondary mutations that impair the efficacy of PI3K inhibitors. These agents can be designed to specifically target and inhibit variants of PIK3CA and other key components of the PI3K signaling pathway, thereby increasing the sensitivity of cancer cells to PI3K inhibitors.

Additionally, drugs can be designed to restore the function of, or mimic the action of, negative regulators that are lost or inactivated during therapy. For example, if PTEN is lost, new drugs can be developed to restore PTEN expression or enhance its tumor suppressor activity. These agents can sensitize cancer cells to PI3K inhibitors and overcome resistance by restoring balance in the PI3K signaling pathway.

Another strategy involves developing drugs that specifically target and inhibit compensatory pathways that are activated in response to treatment. For example, drugs can be designed to selectively block the MAPK [256], or mTOR [257, 258] signaling pathways, which are often upregulated in response to PI3K inhibition. By inhibiting these compensatory pathways, these novel agents may prevent their activation and enhance the efficacy of therapies targeting the PI3K pathway.

Additionally, novel drugs can be developed that target specific signaling nodes or molecular targets important for resistance development. A thorough understanding of the mechanisms underlying resistance will allow researchers to identify points of weakness within signaling pathways and design drugs that selectively inhibit these targets, thereby reducing resistance.

#### Biomarker-guided therapy

Biomarker-based therapy has shown promise to optimise outcomes for cancer patients receiving PI3K inhibitors [259]. By identifying predictive biomarkers that can stratify patients based on their likelihood of responding to PI3K inhibitors, individualized treatment strategies can be tailored to individual profiles, resulting in improved treatment efficacy.

An important aspect of biomarker-based therapy is the identification of specific biomarkers that can reliably predict a patient's response to PI3K inhibitors. These biomarkers can be genetic, molecular, or cellular traits associated with sensitivity or resistance to PI3K inhibition. Researchers can identify and validate these biomarkers by analyzing tumor samples or performing non-invasive tests such as liquid biopsies. For example, genetic alterations in the PIK3CA gene [260, 261], which encodes the PI3K catalytic subunit, have been identified as potential predictive biomarkers. Patients with certain PIK3CA mutations may show an increased response to PI3K inhibitors. Therefore, biomarker-based therapy could identify these mutations through genetic testing and select patients with PIK3CA mutations as good candidates for PI3K inhibitor therapy.

In addition to genetic biomarkers, other molecular markers or changes in signaling pathways may also serve as predictive biomarkers. For example, altered expression or activity of PTEN, which negatively regulates PI3K signaling [262], could influence responses to PI3K inhibitors. Biomarker-based therapy can assess PTEN status through immunohistochemistry or molecular testing to identify patients who may benefit from PI3K inhibitor therapy.

Biomarker-based therapies play a crucial role in tailoring PI3K inhibitor treatments. Identifying biomarkers associated with compensatory pathway activation, such as MAPK and mTOR, can guide combination therapies. Positive biomarker profiles indicate patients likely to respond to PI3K inhibitors, while negative profiles suggest alternative treatment options or combination therapies targeting alternative pathways. This individualized approach optimizes therapeutic response and minimizes side effects, advancing precision oncology in PI3K-associated cancers.

#### Adaptive treatment approaches

Adaptive therapeutic approaches have emerged as dynamic strategies to optimize therapeutic efficacy and overcome resistance in cancer patients [263]. By continuously monitoring treatment response and adjusting treatment based on real-time patient-specific data, these approaches can enable personalized and dynamic adjustments to improve outcomes [264].

Central to the adaptive treatment approach is regular monitoring of treatment response using various techniques such as imaging [265], and liquid biopsy [266]. These monitoring methods provide real-time insight into a patient's tumor dynamics, allowing physicians to assess the efficacy of ongoing therapy.

Treatment strategies can be adjusted to optimize treatment efficacy based on the data collected. For example, if initial treatment with a PI3K inhibitor produces a suboptimal response or demonstrates the emergence of resistance, an adaptive approach allows for timely changes. This may include switching to an alternative PI3K inhibitor with a different binding profile or choosing a combination therapy targeting compensatory pathways identified through real-time monitoring.

Adaptive approaches also include changing treatment schedules and dosages [267]. By fine-tuning treatment regimens based on patient response and tolerability, efficacy can be maximized while minimizing toxicity [268]. For example, doses may be adjusted or treatment interrupted to manage side effects or improve drug delivery. Additionally, adaptive treatment approaches integrate the use of predictive models and algorithms to guide decision making. Combining patient-specific data such as molecular profiling, treatment history and clinical parameters with computational analysis, these models can generate individualized treatment response predictions and identify potential resistance mechanisms. Such predictive modeling enables proactive decision-making, allowing physicians to anticipate and counteract resistance before it becomes clinically apparent.

Adopting adaptive therapeutic approaches in PI3K-associated cancers requires collaboration among clinicians, researchers, and data analysts. Establishing a robust data infrastructure and clinical guidelines enables real-time collection and analysis of patient-specific data, facilitating treatment adjustments based on individual needs. This dynamic approach improves outcomes by allowing timely intervention, early resistance detection, and optimized treatment strategies.

### Combination therapy

Combining PI3K inhibitors with other targeted agents enables a synergistic approach to simultaneously target multiple signaling pathways and address potential resistance mechanisms. Several targeted agents have shown promise in combination with his PI3K inhibitors in the treatment of breast cancer [84].

#### HER2-targeted therapies

In HER2 BC, PI3K signaling is often co-activated with HER2 signaling, which contributes to resistance and poor response to HER2-targeted therapy [269]. To meet this challenge, combinations of PI3K inhibitors and HER2-targeting agents, such as trastuzumab [270, 271], and lapatinib [272, 273], have emerged as promising therapies.

The combination of HER2-targeted therapy and PI3K inhibition in HER2+ BC aims to suppress proliferative signals from HER2 and inhibit the PI3K signaling pathway. This approach enhances anti-tumor activity and improves patient outcomes by overcoming resistance mechanisms and interfering with compensatory signaling.

In addition, this combined strategy offers the potential for synergistic effects, as the PI3K signaling pathway can regulate multiple cellular processes such as cell cycle progression, survival and metabolism that influence tumor growth and therapeutic response [274]. By blocking both pathways, combination therapy aims to achieve a more comprehensive inhibition of tumor cell signaling and proliferation. Pre-clinical trials have shown encouraging results that the combination of PI3K inhibitors and HER2-targeted agents improves objective response rates and progression-free survival in patients with HER2+ BC [275]. These results support the concept of dual pathway inhibition as a powerful strategy to overcome resistance and improve patient outcomes.

Overall, the combination of PI3K inhibitors and HER2-targeted therapy represents a rational and targeted therapeutic approach for HER2+ BC, addressing co-activation of PI3K signaling pathways, overcoming resistance mechanisms, and exerting antitumor efficacy offers the possibility of strengthening. This ultimately provides new opportunities to improve therapeutic efficacy in this specific subtype of breast cancer.

#### CDK4/6 inhibitors

CDK4/6 inhibitors, including Palbociclib [276, 277], ribociclib [278, 279], and abemaciclib [280, 281], have demonstrated notable effectiveness in HR+ BC. These inhibitors work by targeting CDK4/6, inducing cell cycle arrest and suppressing tumor cell proliferation.

A combination of CDK4/6 inhibitors and PI3K inhibitors has proven to be a promising therapeutic strategy. Preclinical and clinical studies have provided evidence of synergy when these two inhibitors are used together [282]. The combination of PI3K inhibitors and CDK4/6 inhibitors in HR+ breast cancer is driven by their complementary mechanisms. PI3K inhibitors suppress cell survival and proliferation by targeting the PI3K signaling pathway, while CDK4/6 inhibitors directly block cell cycle progression at the G1/S phase transition by inhibiting Rb phosphorylation.

Combining these inhibitors may enhance inhibition of tumor growth. This combination synergistically disrupts both the cell cycle and PI3K signaling pathways, resulting in a more comprehensive inhibition of tumor cell proliferation. This approach addresses potential resistance mechanisms associated with single-pathway targeting and has broad-spectrum effects on tumor growth control [283].

Clinical trials investigating the combination of CDK4/6 inhibitors and PI3K inhibitors in HR+ BC have demonstrated promising results, including longer progression-free survival, improved objective response rate, and longer duration of response compared to monotherapy. This combination therapy offers potential advantages in terms of minimizing treatment resistance and optimizing patient outcomes by targeting multiple key pathways involved in tumor growth and survival, overcoming resistance mechanisms that may arise during monotherapy.

#### Endocrine therapies

Endocrine therapies such as aromatase inhibitors [284, 285], and selective estrogen receptor modulators are important treatments for HR+ BC. However, the interplay between PI3K signaling and estrogen receptor signaling may lead to resistance to endocrine therapy. In order to overcome resistance mechanisms and improve treatment response in HR+ BC, the combination of PI3K inhibitors and endocrine therapy has been explored as a potential approach [117].

The PI3K signaling pathway interacts with estrogen receptor signaling to promote cell proliferation and survival [286]. Dysregulation of PI3K signaling may contribute to endocrine resistance by evading or interfering with the effects of endocrine therapy [17]. Combining PI3K inhibitors with endocrine therapy can simultaneously target both signalling pathways, resulting in more comprehensive tumour growth inhibition. PI3K inhibitors block the PI3K signaling pathway, whereas endocrine therapy interferes with estrogen receptor signaling and globally impedes cancer cell survival and proliferation [17].

Preclinical and clinical studies have demonstrated promising results with the combination of PI3K inhibitors and endocrine therapy in HR+ BC. This approach leads to increased tumor growth suppression, improved response rates, and longer progression-free survival compared to endocrine therapy alone [287]. By targeting multiple signaling pathways, this combination strategy overcomes acquired resistance and prevents the emergence of resistance, addressing the complexity of HR+ BC and offering potential for optimized treatment outcomes.

#### mTOR inhibitors

The mTOR is closely related to the PI3K signaling pathway and is central in regulating cell growth and proliferation [288]. The PI3K and mTOR pathways share some points of interaction and co-regulation. Dysregulation of these pathways can lead to increased cell survival, increased cell proliferation, and resistance to therapy [114]. Targeting both pathways simultaneously can achieve a more comprehensive inhibition of tumor growth. The combination of PI3K and mTOR inhibitors represents a dual blockade approach that effectively suppresses signaling cascades in promoting cancer cell growth. This combination inhibits PI3K-mediated activation of mTOR and downstream effectors, thereby synergizing tumor cells [289].

The combination of PI3K inhibitors and mTOR inhibitors has demonstrated encouraging outcomes in cancer treatment. Compared to monotherapy, this synergistic approach enhances progression-free survival, objective response rate, and overall survival. This strategy effectively tackles resistance mechanisms by concurrently targeting the PI3K and mTOR pathways, offering a compelling therapeutic option for patients with this specific type of BC.

#### MEK inhibitors

Activation of the MAPK, particularly by the downstream kinase MEK, may serve as a compensatory mechanism in response to PI3K inhibition [290]. The PI3K and MAPK signaling pathways are interrelated and often activated in cancer cells [291]. When PI3K signaling is inhibited, cancer cells activate MAPK signaling as an alternative survival mechanism, promoting cell proliferation and therapy resistance.

The combination of PI3K and MEK inhibitors represents a dual inhibitory strategy that simultaneously targets the PI3K and MAPK pathways [292, 293]. By blocking both pathways, this combined approach aims to achieve broader inhibition of tumor cell proliferation and survival.

Preclinical studies have shown that the combination of PI3K inhibitors and MEK inhibitors may provide synergistic effects and increase anti-tumor activity compared to monotherapy. This synergistic effect results from the complementary actions of these inhibitors, jointly repressing key signaling nodes in both signaling pathways and impeding compensatory signaling [294].

This combination strategy may overcome resistance mechanisms that may arise during treatment with PI3K inhibitors by simultaneously targeting the PI3K and MAPK signaling pathways. This interferes with compensatory activation of the MAPK pathway, which may contribute to treatment resistance, and improves overall therapeutic response.

## Clinical trials

Several trials have been completed, and some are currently underway to investigate the targeting of the PI3K pathway in breast cancer (Table 6). The ongoing trials are discussed below. The clinical trial (NCT03962647) is a neoadjuvant study investigating the feasibility and tolerability of a 2-week ketogenic diet combined with letrozole in patients with operable early-stage ER+ breast cancer. The primary objective is to assess the viability and tolerability of the dietary intervention alongside endocrine therapy before surgery. Secondary goals include evaluating the combined impact on inhibiting cancer cell growth, measuring insulin/PI3K pathway activation, assessing changes in weight and body composition, and determining the efficacy of inducing a ketogenic state in synergy with endocrine therapy. Participants undergo baseline measurements, initiate a 2-week ketogenic diet with meal replacement shakes, and receive letrozole. Post-intervention, metabolic parameters are re-evaluated, and patients proceed to surgical intervention. The study represents a crucial step in understanding the potential benefits of combining dietary modification and endocrine therapy in ER+ breast cancer treatment [295].

**Table 6:** Clinical trials for targeting the PI3k signalling in breast cancer

Clinical trial	Drug/Treatment	Study type and phase	Enrollment	Study starts and completion year	Status
NCT02506556	BYl719	II (Interventional)	36	2015–2021	Completed
NCT01513356	BKM120	I (Interventional)	20	2012–2014	Completed
NCT00499603	Paclitaxel 5-Fluorouracil Epirubicin Cyclophosphamide RAD001	II (Interventional)	62	2007–2017	Unknown
NCT03962647	Letrozole 2-Week Ketogenic Diet	I (Interventional)	31	2019–2025	Active (Not recruiting)
NCT01339442	BKM120 Fulvestrant Biopsy	I (Interventional)	31	2011–2016	Completed
NCT02058381	BYL719 BKM120	I (Interventional)	40	2014–2018	Completed
NCT01363232	BKM120 MEK162	I (Interventional)	89	2011–2017	Completed
NCT01337765	BEZ235 MEK162	I (Interventional)	29	2011–2013	Completed
NCT01155453	BKM120 GSK1120212	I (Interventional)	113	2010–2014	Completed
NCT01920061	Gedatolisib` Docetaxel Cisplatin Dacomitinib	I (Interventional)	110	2013–2020	Completed
NCT01980277	LY2780301 paclitaxel	I, II (Interventional)	68	2014–2018	Terminated
NCT05810870	MEN1611 Eribulin	II (Interventional)	28	2023–2027	Active (Recruiting)
NCT01501604	BKM120	II (Interventional)	0	2012–2013	Withdrawn
NCT04108858	Copanlisib Pertuzumab Trastuzumab	I, II (Interventional)	2	2021–2022	Terminated
NCT02069158	PF-05212384 Paclitaxel Carboplatin	I (Interventional)	17	2014–2019	Completed
NCT01298713	Tamoxifen Everolimus	II (Interventional)	111	2008–2018	Completed
NCT04345913	Biopsy Biospecimen Collection Computed Tomography Copanlisib Hydrochloride Eribulin Mesylate Magnetic Resonance Imaging	I, II (Interventional)	106	2021–2023	Active (Not recruiting)
NCT03243331	Gedatolisib PTK7-ADC	I (Interventional)	18	2018–2020	Completed
NCT03765983	Trastuzumab GDC-0084	II (Interventional)	47	2019–2025	Active (Recruiting)

The ongoing SABINA study (NCT05810870) is a Phase II clinical trial designed to assess the safety and efficacy of MEN1611, both as a monotherapy and in combination with eribulin, in patients with unresectable, HR/HER2-negative, PIK3CA/PTEN-altered locally advanced or metastatic breast cancer (MpBC). The trial initiates with a run-in period to evaluate the safety and tolerability of the combination, aiming to establish the dosing schedule based on toxicity profiles identified in a preliminary study. The primary objective is to evaluate the efficacy of the eribulin and MEN1611 combination, measured by clinical benefit rate (CBR) in patients with unresectable MpBC (cohort A). Simultaneously, the study assesses MEN1611's efficacy as monotherapy through objective response rate (ORR) after 6 weeks (cohort B). The study plans to enroll 28 patients, with Cohort A potentially expanding based on a toxicity review. Cohort B is contingent on positive results in Cohort A, utilizing Simon's two-stage minimax design to guide further expansion based on treatment response in a staged manner. This study represents a significant step in understanding the therapeutic potential of MEN1611 in MpBC [296].

Another Phase I/II clinical trial (NCT04345913) investigates the safety, toxicity profile, and efficacy of combining copanlisib hydrochloride and eribulin mesylate for treating metastatic triple-negative breast cancer. Following a prior dose-escalation study, the primary objectives are to determine safety parameters and compare progression-free survival (PFS) between patients treated with eribulin monotherapy and those receiving the combination. Secondary objectives include evaluating objective response rate (ORR), clinical benefit rate (CBR), and safety in both arms, along with documenting antitumor activity. The study aims to assess outcomes based on PIK3CA/PTEN pathway alterations and employs biomarkers to evaluate targeted inhibition by copanlisib and eribulin. Patient randomization into two groups involves different administration schedules, and both groups undergo imaging, biopsies, and blood sample collections at specified intervals. Follow-up occurs every 3 months post-treatment for up to 36 months. Overall, this study is crucial for understanding the potential therapeutic benefits of copanlisib and eribulin in the context of metastatic triple-negative breast cancer [297].

The ongoing Phase II clinical trial (NCT03765983) investigates the investigational intervention GDC-0084 in combination with the FDA-approved targeted therapy trastuzumab for the treatment of HER2-positive breast cancer, specifically focusing on patients with brain metastases. GDC-0084, though not FDA-approved, exhibits the capability to inhibit PI3 kinase, disrupting a crucial pathway in cancer cell growth. In contrast, trastuzumab, an established HER2-targeted therapy, disrupts proliferative signals and enhances immune recognition of cancer cells. The study aims to unravel the potential synergistic effects of combining GDC-0084 with trastuzumab, shedding light on the responses of HER2-positive breast cancers. The trial represents a critical milestone in understanding the therapeutic benefits of this combination in the challenging context of HER2-positive breast cancer with brain metastases [298].

## Conclusion

In summary, advances in predictive, preventive and personalized medicine and our understanding of the challenges and opportunities in developing PI3K inhibitors for BC have paved the way for significant progress in this field. By focusing on predictive biomarkers, preclinical models, and genomic approaches, researchers have made significant progress in identifying biomarkers that can help predict response to PI3K inhibitors in BC patients. These predictive biomarkers provide valuable insight into patient stratification and individualized treatment decisions.

Moreover, preventive medicine strategies for PI3K activation in BC have shown promise. Preclinical evidence supports the protective effect of PI3K inhibitors, raising hopes for BC risk reduction in high-risk populations. Understanding the mechanisms underlying these preventive effects may open up the possibility of developing new preventive interventions.

Personalized medicine approaches have revolutionized BC treatment by addressing multiple factors that influence treatment response, including tumor stage, molecular subtype, and patient-specific characteristics. Clinical evidence has demonstrated the efficacy of PI3K inhibitors in specific patient subgroups, allowing targeted therapy to be tailored to individual patients. Overcoming resistance to PI3K inhibitors remains a challenge, but continued efforts to identify mechanisms of resistance and develop combination therapies promise better outcomes.

Finally, PI3K inhibitor development for BC needs to address its toxicity and side effect profile. Rigorous evaluation and monitoring of adverse events are critical to optimize patient compliance and adherence to treatment. Additionally, combining PI3K inhibitors with other targeted agents, such as his HER2-targeted therapy and CDK4/6 inhibitors, offers the opportunity to improve therapeutic efficacy and overcome resistance mechanisms.

Overall, a thorough understanding of the challenges and opportunities in developing PI3K inhibitors for BC, as well as integrating predictive, preventive, and personalized medicine approaches, has the potential to transform patient care. Harnessing the power of predictive biomarkers, preventive strategies, individual treatment options, and combination therapies to maximize treatment efficacy, improve patient outcomes, and ultimately advance the therapeutic field of BC.

## Future directions

In the field of predictive medicine, future research should focus on identifying and validating predictive biomarkers for PI3K inhibitors in BC. This requires the study of specific genetic alterations, protein expression patterns and molecular signatures that can accurately predict treatment response. Furthermore, developing and refining preclinical models of PI3K inhibitors in BC will improve their predictive value and enable better transition to clinical trials. Incorporating patient-derived models, organoids, and 3D culture systems can provide a more clinically relevant understanding of drug efficacy and resistance mechanisms. Genomic approaches such as next-generation sequencing and multi-omics analysis should be used to comprehensively identify potential biomarkers of response to PI3K inhibitors. Integrating genomic data with clinical outcomes will improve our understanding of patient stratification and serve as the basis for personalized treatment decisions.

In the field of preventive medicine, prospective clinical trials are needed to evaluate the preventive efficacy of PI3K inhibitors in high-risk populations, such as those with a family history of BC or precancerous lesions. Long-term follow-up will provide insight into prophylactic interventions' sustained benefits and safety profile. Further research into the underlying mechanisms of the preventive effects of PI3K inhibitors is important. Elucidating how PI3K activation contributes to BC development and progression will help identify new targets and avenues for prevention strategies. Combinatorial approaches targeting both PI3K and other relevant signaling pathways involved in BC development should be explored to improve preventive efficacy. Understanding the interactions between different signaling networks will facilitate the development of more comprehensive preventative measures.

In the field of personalized medicine, refinement of patient selection criteria based on tumor stage, molecular subtype and other factors will optimize the use of PI3K inhibitors in BC treatment. Prospective studies should validate the clinical utility of these selection criteria and investigate additional factors influencing treatment response, such as the TME and host genetic variation. Examining the efficacy of PI3K inhibitors in specific patient subgroups, such as those with different genetic mutations or resistance mechanisms, will guide individualized therapeutic strategies. Prospective clinical trials using stratified patient cohorts are needed to identify patients who will benefit most from PI3K inhibitors. Overcoming resistance to PI3K inhibitors is an important research area. Combination therapies targeting resistance mechanisms such as MAPK signaling or alternative signaling pathways should be considered. Developing new drugs and rational combinations based on understanding resistance mechanisms will help improve treatment outcomes.

Comprehensive assessment and treatment of the toxicity and side effect profiles associated with these inhibitors should be a priority when addressing the challenges and opportunities in the development of PI3K inhibitors for BC. Identifying strategies that reduce toxicity and optimize patient compliance improve treatment adherence and quality of life. Further elucidation of the mechanisms of resistance to PI3K inhibitors is needed. This includes understanding adaptive feedback loops, genetic alterations, and other molecular events that produce resistance. Combination therapies that target specific resistance mechanisms or use synergistic drug combinations show promise in overcoming resistance. Further research is needed into combination therapy approaches using PI3K inhibitors and other targeted agents, such as HER2-targeted therapies and immune checkpoint inhibitors. Rational design of combination therapies based on mechanistic insights and preclinical evidence will improve therapeutic efficacy and expand treatment options.

In summary, future directions in the field of PI3K inhibitors for BC include improving predictive medicine approaches, developing prevention strategies, advancing personalized medicine approaches, and addressing drug development challenges and opportunities. These efforts will streamline treatment selection, improve preventive interventions, improve patient outcomes, and bring us closer to the realization of precision medicine in breast cancer.

## Data Availability

Not applicable.

## Abbreviations

BC: Breast cancer
PI3K: Phosphatidylinositol-3-kinase
EGFR: Epidermal growth factor receptor
IGFR: Insulin-like growth factor receptor
HER2: Human epidermal growth factor receptor 2
PIP2: Phosphatidylinositol 4,5-bisphosphate
PIP3: Phosphatidylinositol 3,4,5-trisphosphate
pAKT: Phosphorylated AKT
AKT1: V-AKT Murine Thymoma Viral Oncogene Homolog 1
PIK3R1: Phosphoinositide 3-kinase regulatory subunit 1
INPP4B: Inositol polyphosphate 4-phosphatase type II
ctDNA: Circulating tumor DNA
CTCs: Circulating tumor cells
ER: Estrogen receptor
PDX: Patient-derived xenografts
GEMM: Genetically engineered mouse model
mTOR: Mammalian target of rapamycin
VEGF: Vascular endothelial growth factor
NK: Natural killer
Treg: Regulatory T cells
HR+: Hormone receptor-positive
TNBC: Triple-negative breast cancer
MAPK: Mitogen-activated protein kinase
